# Protective Efficacy of Passive Immunization with Monoclonal Antibodies in Animal Models of H5N1 Highly Pathogenic Avian Influenza Virus Infection

**DOI:** 10.1371/journal.ppat.1004192

**Published:** 2014-06-12

**Authors:** Yasushi Itoh, Reiko Yoshida, Shintaro Shichinohe, Megumi Higuchi, Hirohito Ishigaki, Misako Nakayama, Van Loi Pham, Hideaki Ishida, Mitsutaka Kitano, Masahiko Arikata, Naoko Kitagawa, Yachiyo Mitsuishi, Kazumasa Ogasawara, Hideaki Tsuchiya, Takahiro Hiono, Masatoshi Okamatsu, Yoshihiro Sakoda, Hiroshi Kida, Mutsumi Ito, Le Quynh Mai, Yoshihiro Kawaoka, Hiroko Miyamoto, Mari Ishijima, Manabu Igarashi, Yasuhiko Suzuki, Ayato Takada

**Affiliations:** 1 Department of Pathology, Shiga University of Medical Science, Otsu, Shiga, Japan; 2 Division of Global Epidemiology, Hokkaido University Research Center for Zoonosis Control, Sapporo, Japan; 3 Laboratory of Microbiology, Department of Disease Control, Graduate School of Veterinary Medicine, Hokkaido University, Sapporo, Japan; 4 Infectious Diseases, Medicinal Research Laboratories, Shionogi & Co., Ltd., Toyonaka, Osaka, Japan; 5 Research Center for Animal Life Science, Shiga University of Medical Science, Otsu, Shiga, Japan; 6 Division of Virology, Department of Microbiology and Immunology, Institute of Medical Science, University of Tokyo, Tokyo, Japan; 7 National Institute of Hygiene and Epidemiology, Hanoi, Vietnam; 8 Department of Pathobiological Sciences, School of Veterinary Medicine, University of Wisconsin, Madison, Wisconsin, United States of America; 9 Division of Bioinformatics, Hokkaido University Research Center for Zoonosis Control, Sapporo, Japan; Johns Hopkins University - Bloomberg School of Public Health, United States of America

## Abstract

Highly pathogenic avian influenza (HPAI) viruses of the H5N1 subtype often cause severe pneumonia and multiple organ failure in humans, with reported case fatality rates of more than 60%. To develop a clinical antibody therapy, we generated a human-mouse chimeric monoclonal antibody (MAb) ch61 that showed strong neutralizing activity against H5N1 HPAI viruses isolated from humans and evaluated its protective potential in mouse and nonhuman primate models of H5N1 HPAI virus infections. Passive immunization with MAb ch61 one day before or after challenge with a lethal dose of the virus completely protected mice, and partial protection was achieved when mice were treated 3 days after the challenge. In a cynomolgus macaque model, reduced viral loads and partial protection against lethal infection were observed in macaques treated with MAb ch61 intravenously one and three days after challenge. Protective effects were also noted in macaques under immunosuppression. Though mutant viruses escaping from neutralization by MAb ch61 were recovered from macaques treated with this MAb alone, combined treatment with MAb ch61 and peramivir reduced the emergence of escape mutants. Our results indicate that antibody therapy might be beneficial in reducing viral loads and delaying disease progression during H5N1 HPAI virus infection in clinical cases and combined treatment with other antiviral compounds should improve the protective effects of antibody therapy against H5N1 HPAI virus infection.

## Author Summary

The H5N1 highly pathogenic avian influenza virus has been circulating in poultry in Asia, the Middle East, and Africa since its first appearance in southern China in 1996. This virus occasionally infects humans with a high case mortality rate and poses a significant pandemic threat. Since neutralizing antibodies generally play a major role in protective immunity against influenza viruses, antibody therapy is a potential option for preventing highly lethal infection with the H5N1 virus in humans. Here we evaluated the protective potential of a human-mouse chimeric monoclonal antibody with strong neutralizing activity against H5N1 viruses in mouse and nonhuman primate models of lethal H5N1 virus infection. The therapeutic use of the neutralizing antibody resulted in reduced viral loads and improved survival in animals infected with highly pathogenic H5N1 viruses. It was noted that the protective effects were more prominent in immunosuppressed macaques, which might provide a model of protection against severe clinical disease in immunocompromised patients. In addition, combination therapy together with an antiviral drug reduced the selection of escape mutants. Collectively, this study suggests that antibody therapy may have beneficial effects in clinical cases of H5N1 HPAI virus infection in humans.

## Introduction

Influenza A viruses are divided into subtypes based on the antigenicity of two envelope glycoproteins, hemagglutinin (HA) and neuraminidase (NA). To date, H1-H16 HA and N1-N9 NA subtypes have been found in wild aquatic birds, the natural reservoir of influenza viruses [1]–[3]. Of these HA subtypes, only some avian influenza viruses of the H5 and H7 subtypes are known to become highly pathogenic avian influenza (HPAI) viruses under natural conditions. While HPAI viruses cause an acute systemic disease in poultry with a mortality rate that often approaches 100%, avian to human transmission of HPAI viruses is limited and HPAI viruses had never been reported to cause lethal infection in humans until the first emergence of an H5N1 HPAI virus in southern China in 1996.

The H5N1 HPAI virus has been circulating in poultry for more than a decade since its reemergence in southern China in 2003, and has caused unprecedented outbreaks in wild birds and poultry in Asia, the Middle East, and Africa [4]–[10]. The H5N1 HPAI virus occasionally infects humans with a high case mortality rate and poses a significant pandemic threat [11], [12], [13]. Since 2003, 641 laboratory-confirmed human cases of H5N1 HPAI virus infection have been reported from 15 countries, with 380 fatal cases (as of October 8, 2013) [12]. In fact, prior to the emergence of the swine-origin H1N1 pandemic virus in 2009, the impact on animal and public health of the Asian origin H5N1 HPAI virus led to the prediction that a virus of the H5 subtype might cause the next pandemic, since this HA subtype is distinct from those of viruses circulating in the human population (i.e., subtypes H1 and H3) [13].

In recent years, passive immunization with human or humanized monoclonal antibodies (MAbs) specific to viral proteins has been tested in animal models and clinical trials, providing evidence of the effectiveness of MAbs for prophylaxis or treatment of infectious diseases [14]. Indeed, a humanized MAb specific to Respiratory syncytial virus F protein is already approved by the US Food and Drug Administration and used in clinical cases. Importantly, particular attention has been paid to antibody therapy against highly lethal diseases such as rabies [15]–[17], severe acute respiratory syndrome [18], [19], Hendra [20], Nipah [21], and Ebola viruses [22]–[25].

It is known that HA, which is responsible for both receptor binding and fusion of the virus envelope with the host cell membrane, is the primary target of neutralizing antibodies against influenza viruses. Since antibodies generally play a major role in protective immunity against influenza virus infection [26], antibody therapy might be a potential option for preventing lethal infection of humans by the H5N1 HPAI virus. In this study, we genetically modified a mouse MAb (m61) neutralizing the infectivity of H5N1 HPAI viruses to create human-mouse chimeric MAb (ch61), aiming at clinical application, and evaluated its protective potential in mouse and nonhuman primate models of H5N1 HPAI virus infection.

## Materials and Methods

### Viruses and cells

HPAI virus strains A/Hong Kong/483/1997 (H5N1) (HK483), A/Viet Nam/1194/2004 (H5N1) (VN1194), and A/Vietnam/UT3040/2004 (H5N1) (VN3040) from the repository of our laboratory, were propagated in Madin-Darby canine kidney (MDCK) cells from the repository of our laboratory and stored at −80°C until use. HK483, VN1194, and VN3040 belong to clades 0, 1, and 1 in a phylogenetic tree, respectively [27]. MDCK cells were grown in Eagle's minimal essential medium supplemented with 10% calf serum. All experiments using infectious viruses were performed in the biosafety level 3 facilities of the Hokkaido University Research Center for Zoonosis Control and Research Center for Animal Life Science, Shiga University of Medical Science.

### Generation of mouse monoclonal antibodies

Mouse MAb 61-2-1 (m61), was generated according to standard procedures. Briefly, six-week-old female BALB/c mice (Japan SLC) were immunized intramuscularly two times with 100 µg of formalin-inactivated purified virions and boosted intraperitoneally [23]. Spleen cells harvested 3 days after boosting were fused to P3U1 myeloma cells according to standard procedures. Hybridomas were screened for secretion of HA-specific MAbs by enzyme-linked immunosorbent assay (ELISA), and cloned by limiting dilution. The resulting cell clones were inoculated into BALB/c mice intraperitoneally to produce ascites. Antibodies were purified from ascites using the Affi-Gel Protein A MAPS II Kit (Bio-Rad). Mouse MAbs ZGP133 and ZGP226 used as control antibodies were generated as described previously [23].

### Generation of human-mouse chimeric monoclonal antibodies

Human-mouse chimeric MAb ch61 was generated and purified from culture supernatants as described previously [23]. Briefly, total RNA was extracted from mouse hybridoma cells producing MAb m61, and the variable heavy- and light-chain regions were amplified by RT-PCR with primers designed for the antibodies. The PCR products were cloned into an expression vector. Stable cell lines expressing recombinant MAb ch61 were obtained by transfection of CHO DG44 cells (Invitrogen, Carlsbad, CA). Chimeric MAbs (ch133 and ch226) specific for the Ebola virus glycoprotein were generated as control MAbs using the same methodology [23]. These human-mouse chimeric MAbs were purified from culture supernatants using rProtein A Sepharose Fast Flow (GE Healthcare) and EndoTrap red (Profos AG). MAb purity (>98%) and endotoxin levels (<1.0 EU/ml) were confirmed by performing SDS-PAGE and with an Endospecy ES-50M kit (Seikagaku Corporation), respectively.

### Neutralization assay

Serially diluted antibodies (100 µl) were mixed with 200 plaque forming units (PFU) of H5N1 viruses for 1 h at room temperature, and inoculated onto MDCK cells. After 1 h, the inoculum was removed and the cells were overlaid with 1% Bacto-Agar (BD) in Eagle's minimal essential medium (MEM). Two days later, the number of plaques was counted and the percentage of plaque reduction was calculated.

### Selection of escape mutants *in vitro*

Escape mutants were selected by culturing VN1194 in MDCK cells in the presence of MAb m61. Serial dilutions of VN1194 were mixed with purified MAb m61 (final concentration of 10 µg/ml), incubated for 1 h, and the mixtures were inoculated into confluent MDCK cells in 6-well tissue culture plates. After 1 h adsorption, the cells were overlaid with MEM containing 1% agar and MAb m61 ascites (final dilution of 1∶1000), and then incubated for 2 days at 35°C. Eight escape mutants were purified from single isolated plaques, and propagated in MDCK cells with serum-free MEM containing trypsin. The nucleotide sequences of the HA genes of the parent strains and the escape mutants were determined and the deduced amino acid sequences were compared among these viruses (H3 numbering).

### Passive immunization and protection tests of mice

Six-week-old female BALB/c mice were passively immunized by intraperitoneal injection with 200 µg of purified MAbs m61 or ch61 24 hours before, or 24 hours or 72 hours after intranasal challenge with 50 µl of 12.5×50% mouse lethal dose of HK483 under anesthesia with isoflurane. Control groups were administered with control antibodies (mixture of MAbs ZGP133/ZGP226 or ch133/ch226) or phosphate-buffered saline (PBS). Animals were monitored daily for weight loss and clinical signs. Five days after the challenge, mice were euthanized to obtain lung tissue samples. Lung homogenates (10% w/v) prepared in MEM were centrifuged at 3,000× g for 10 min, and then the supernatants were examined for virus infectivity. Virus titers were measured by a plaque assay using MDCK cells.

### Preparation of nonhuman primate study

The animal experiments were conducted in strict compliance with animal husbandry and welfare regulations. Food pellets of CMK-2 (CLEA Japan) provided once a day after recovery from anesthesia and drinking water were available *ad libitum*. Animals were singly housed in the cages equipping bars to climb up and puzzle feeders for environmental enrichment under controlled conditions of humidity (60±5%), temperature (24±1°C), and light (12 h light/12 h dark cycle, lights on at 8:00 A.M.). Five- to seven-year-old female cynomolgus macaques (*Macaca fascicularis*) from the Philippines (Ina Research) were used. The cynomolgus macaques used in the present study were healthy adults. The absence of influenza A virus NP-specific antibodies in their sera was confirmed before experiments using an antigen-specific ELISA, AniGen AIV Ab ELISA (Animal Genetics), for currently circulating influenza virus. Three weeks before virus inoculation, a telemetry probe (TA10CTA-D70, Data Sciences International) was implanted in the peritoneal cavity of each macaque under ketamine/xylazine anesthesia followed by isoflurane inhalation to monitor body temperature. The macaques used in this study were free from herpes B virus, hepatitis E virus, *Mycobacterium tuberculosis*, *Shigella* spp., *Salmonella* spp., and *Entamoeba histolytica*. Individual macaques were distinguished by treatments and numbers: C: macaques injected with control MAbs, T: macaques treated with MAb ch61, IC: immunosuppressed macaques injected with control MAbs, IT: immunosuppressed macaques treated with MAb ch61, ICP: immunosuppressed macaques injected with MAbs and peramivir, ITP: immunosuppressed macaques treated with MAb ch61 and peramivir.

### Antibody treatments and protection tests of macaques

Macaques (2.4–3.1 kg) were inoculated (day 0) with VN3040 (total 3×10^6^ PFU/7 ml) in their nasal cavities (0.5 ml for each nostril) and on their tonsils (0.5 ml for each tonsil) with pipettes and into the trachea (5 ml) with catheters under ketamine/xylazine anesthesia. MAb ch61 or control MAbs (a mixture of MAbs ch133 and ch226) were administered intravenously twice (20 mg/head/dose; 6.5–8.3 mg/kg) on days 1 and 3 after infection. Animals were monitored daily (approximately every 12 hours) for clinical scoring (Table S1). Serum samples were obtained on days −1, 1, 3, 5, and 7. For virus titration, cotton sticks (TE8201, Eiken Chemical) were used to collect fluid samples from the nasal cavities and tracheas under ketamine/xylazine anesthesia, and the sticks were subsequently immersed in 1 ml of PBS containing 0.1% bovine serum albumin (BSA) and antibiotics. A bronchoscope (Machida Endoscope) and cytology brushes (Olympus) were used to obtain bronchial samples. The brushes were immersed in 1 ml of PBS with BSA. Viral titers were determined by the tissue culture infectious dose (TCID_50_) in MDCK cells [28]. For immunosuppressive treatments of macaques, cyclophosphamide (CP) (Nacalai Tesque) and cyclosporine A (CA) (Novartis Pharma) were used [29]. CP (40 mg/kg) was administered intravenously by bolus injection on days −7, −5, −3, −1, and 0. CA (50 mg/kg) was administered orally into stomach using a catheter from day −7 to day 6. We confirmed that the treatment with CP and CA decreased the number of white blood cells in the macaques (Fig. S1). In some experiments, peramivir hydrate (30 mg/kg/dose, provided by Shionogi & Co., Ltd.) was administered intravenously by bolus injection once a day from day 1 to day 5 after infection [30]. Since patients with a severe respiratory illness might have a difficulty to intake or inhale drugs, we chose peramivir hydrate as an antiviral agent with intravenous injection. The concentrations of cytokines in sera and tissue homogenates were measured using the Milliplex MAP nonhuman primate cytokine panel and Luminex 200 (Millipore). Although the experiment was originally designed to collect samples from all animals for virology and immunology studies terminating on day 7, some animals were euthanized when their clinical scores reached 15 (a humane endpoint) and subjected to autopsy to collect tissue samples. Macaques that were unfortunately found dead during the intervals of the monitoring time points were also immediately subjected to autopsy. These animals (i.e., euthanized or dead) were counted as nonsurvivors.

### Histological examination

After autopsy on indicated days after virus infection, lung tissue samples were fixed with 10% formalin, and embedded in paraffin. Sections were stained with hematoxylin and eosin (H & E). Influenza virus nucleoprotein (NP) antigens were stained with antisera of rabbits immunized with an NP synthetic peptide (AFTGNTEGRTSDMR at positions 428–441 of the NP sequence: GenBank accession number, ADC34563) after treatment in a pressure cooker in 0.01 M citrate-phosphate buffer. After incubation with anti-rabbit immunoglobulin antibody conjugated with horseradish peroxidase (Nichirei Bioscience Inc.), NP was detected with diaminobenzidine (Nichirei Biosciences Inc.).

### Ethics statement

Animal studies were carried out in strict accordance with the Guidelines for Proper Conduct of Animal Experiments of the Science Council of Japan. The animal experiments were conducted in strict compliance with animal husbandry and welfare regulations. The mouse study was approved by the Hokkaido University Animal Care and Use Committee (Permit number: 08-0234). The nonhuman primate study was also carried out in strict accordance with the Guidelines for the Husbandry and Management of Laboratory Animals of the Research Center for Animal Life Science at Shiga University of Medical Science and Standards Relating to the Care and Management, etc. of Experimental Animals (Notification No. 6, March 27, 1980 of the Prime Minister's Office, Japan). The protocol was approved by the Shiga University of Medical Science Animal Experiment Committee (Permit number: 2011-6-9HHH). All procedures were performed under ketamine and xylazine anesthesia, and all efforts were made to minimize suffering. Regular veterinary care and monitoring, balanced nutrition, and environmental enrichment were provided by the Research Center for Animal Life Science at the Shiga University of Medical Science. Macaques were euthanized at endpoint (7 days after virus inoculation for immunological and virological analysis) using ketamine and xylazine anesthesia followed by intravenous injection of pentobarbital (200 mg/kg). Animals were monitored twice a day during the study to be clinically scored as shown in Table S1. Animals would be euthanized if their clinical scores reached 15 (a humane endpoint).

## Results

### *In vitro* characterization of anti-H5 MAbs m61 and ch61

**Figure 1:**
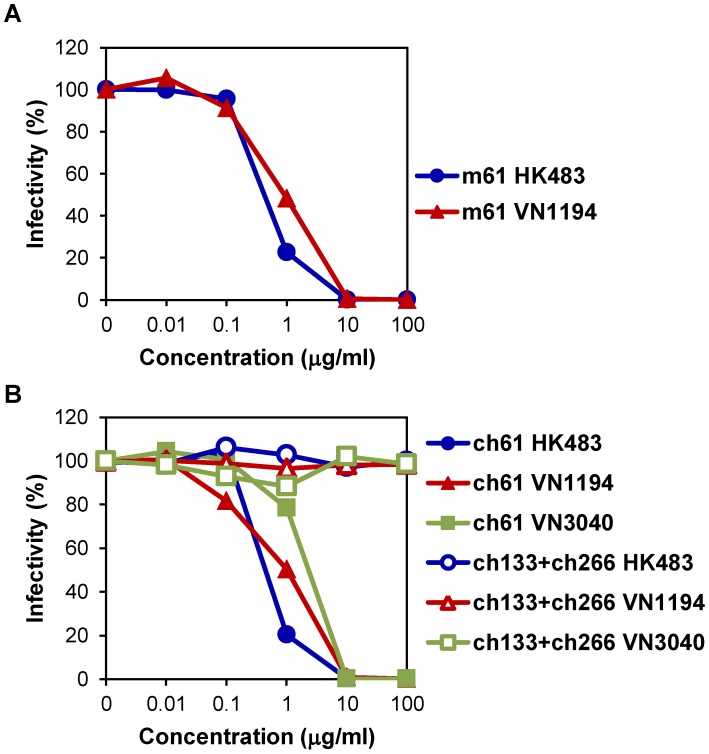
Neutralizing activities of MAbs m61 and ch61 against H5N1 HPAI viruses. Purified MAbs m61 (A) and ch61 (B) at the indicated concentrations were mixed with HK483, VN1194, or VN3040 and inoculated onto MDCK cells. A mixture of MAbs ch133 and ch226 was used as a control. The percentage of infectivity was calculated as follows: Infectivity (%) = the number of plaques with antibody/the number of plaques without antibody ×100. Averages of three independent experiments are shown.

MAb m61 showed neutralizing activities against HK483 and VN1194 (Fig. 1A). The 50% inhibitory concentrations of MAb m61 against HK483 and VN1194 were 0.42 and 0.92 µg/ml, respectively. To determine the epitope for MAb m61, escape mutants of VN1194 were selected in the presence of this MAb and the deduced amino acid sequences of the parent virus and mutants were compared. Lysine to threonine, asparagine, and glutamic acid substitutions were found at position 193 in 12.5, 25.0, and 12.5% of the cloned mutants, respectively, and 50% of the mutants had substitution from lysine to glutamic acid at position 222 (data not shown). The amino acid residue at position 193 is located near the receptor-binding site on the antigenic sites of HA molecules [31]–[34]. Accordingly, MAb m61 showed hemagglutination-inhibition activity (data not shown). We then converted MAb m61 into the human-mouse chimeric MAb ch61, and its neutralizing activities against HK483, VN1194, and VN3040 were analyzed in vitro (Fig. 1B). MAb ch61 significantly reduced the infectivity of these H5N1 viruses in a dose-dependent manner, whereas the negative control MAbs did not. The 50% inhibitory concentrations of MAb ch61 against HK483, VN1194, and VN3040 were 0.43, 1.00, and 2.29 µg/ml, respectively. These values were similar to those of the original mouse MAb m61, indicating that genetic modification of this MAb did not significantly affect the neutralizing activity *in vitro*.

### Protective efficacy of passive immunization with MAbs m61 and ch61 in mice

**Figure 2:**
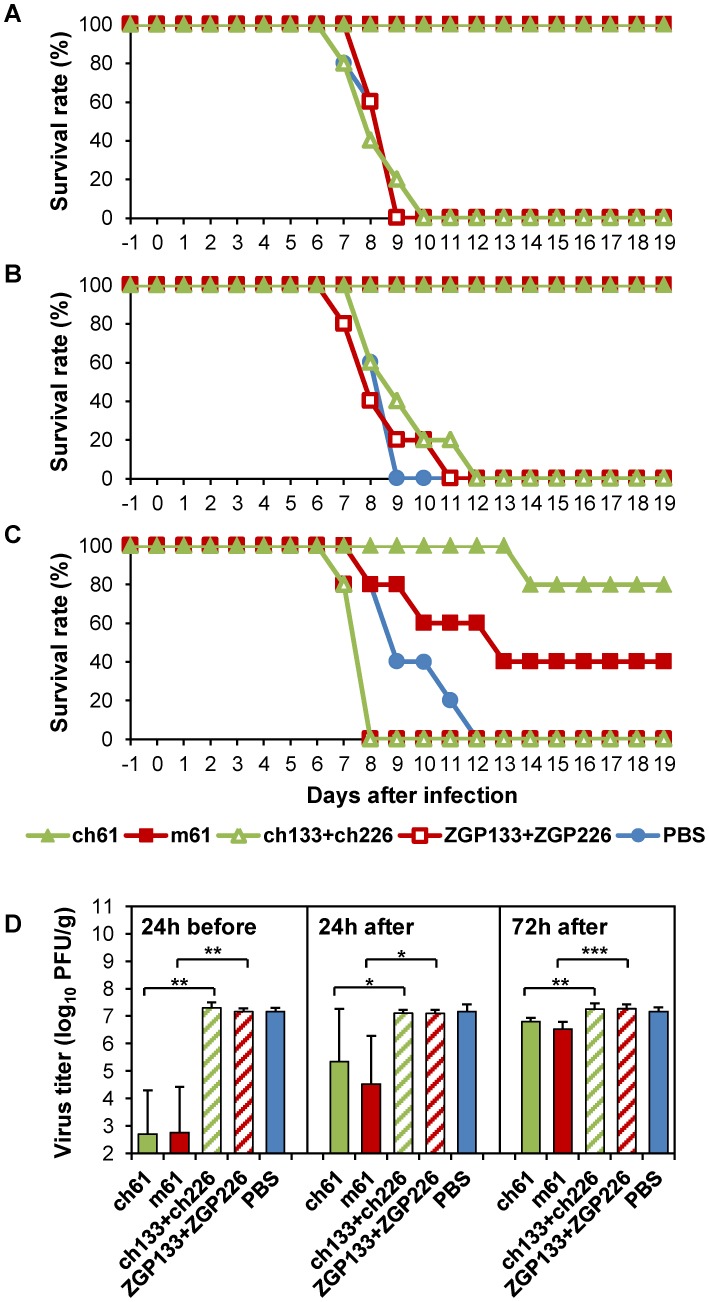
Protective efficacy of passive immunization with MAbs m61 and ch61 against HK483 in a mouse model. Ten mice in each group were treated intraperitoneally with 200 µg of purified MAb m61 or ch61 24 hours before (A), 24 hours after (B) or 72 hours after (C) virus challenge with a lethal dose of HK483 and 5 mice were monitored for their clinical signs/survival and another 5 mice were used for virus titration. Control mice were given control antibodies (ZGP133+ZGP226 or ch133+ch226) or PBS. Five days after the challenge, lung tissue samples were collected to measure virus titers (D). The averages titers and standard deviations of 5 mice are shown. Titers below the limit of detection were assigned a value of 2. Significant differences (Student *t*-test) were indicated by asterisks (*** p<0.001, ** p<0.01, * p<0.05).

We next investigated the potential of MAbs m61 and ch61 to protect mice from infection by HK483, known to be highly virulent for mice [35], [36]. Mice treated with these antibodies 1 day before or 1 day after virus challenge with a lethal dose of HK483 survived without clinical symptoms, whereas all control mice died (or were euthanized) within 9 days after the challenge (Figs. 2A, B). Control mice uniformly showed severe weight loss (>25%) (data not shown). Treatment at 3 days after infection also partially protected the mice (Fig. 2C), although 2 surviving mice treated with m61 showed moderate weight loss (<15%) (data not shown). All control mice exhibited severe weight loss (>25%) and succumbed to HK483 infection. Consistent with the survival data, lung virus titers of mice treated with these anti-H5 HA MAbs 1 day before virus challenge were significantly lower than those of mice given the respective control antibodies (Fig. 2D). While of statistical significance, treatment after infection only modestly reduced the titers (Fig. 2D). These results indicated that MAbs m61 and ch61 were highly protective against H5N1 HPAI virus in mice.

### Efficacy of chimeric anti-H5 MAb ch61 in immunocompetent macaques infected with H5N1 HPAI virus isolated from a human patient

**Figure 3:**
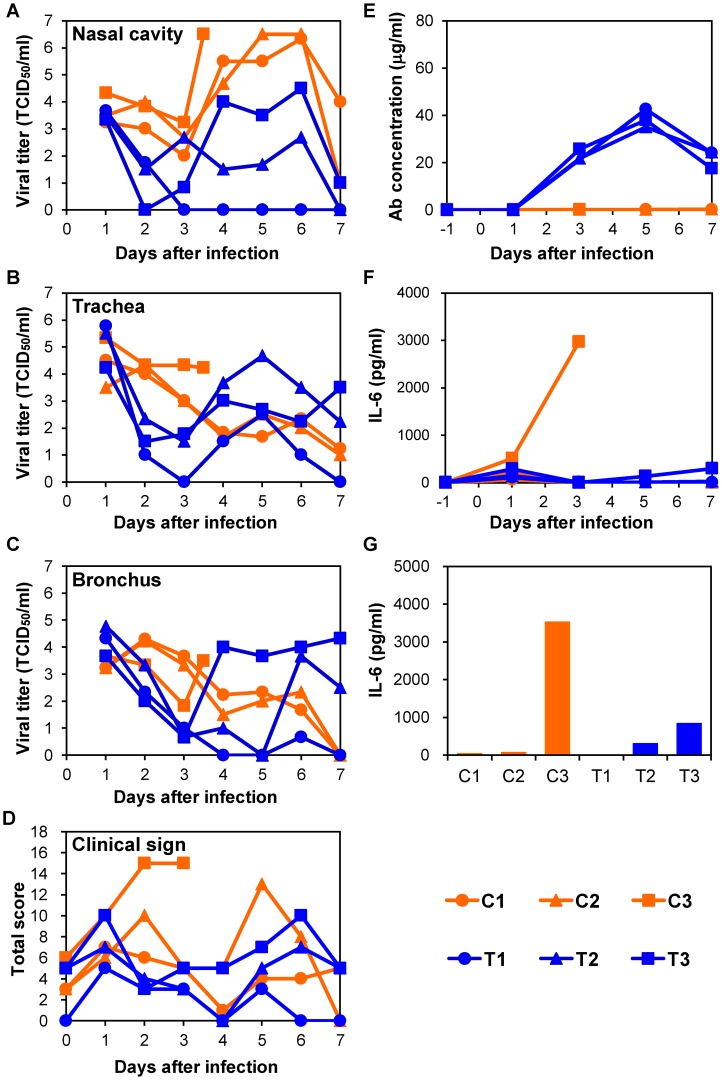
Protection of immunocompetent macaques treated with MAb ch61 against VN3040 infection. Macaques infected with VN3040 (3×10^6^ PFU) on day 0 were injected intravenously with control MAbs (C1–C3, orange) or MAb ch61 (T1–T3, blue) on days 1 and 3. Viral titers in nasal (A), tracheal (B), and bronchial (inside lungs) (C) swab samples were determined using MDCK cells. Viral titers under the detection limit are indicated as 0. Clinical signs were scored with the parameters shown in Table S1 (D). Serum samples were collected from macaques during the period of the experiments and antibodies specific to influenza virus HA were quantified by ELISA [23] (E). IL-6 concentrations in serum samples (F) and lung tissue samples (G) were measured as described in the Materials and Methods section. Lung tissue samples of macaque C3 and other macaques were collected at autopsy on day 3 and on day 7, respectively, and IL-6 concentrations in 10% (w/v) homogenates in saline were measured.

To examine therapeutic efficacy of MAb ch61 in a nonhuman primate model of H5N1 HPAI virus infection, VN3040 was used, since this virus causes severe, often lethal, disease in cynomolgus macaques [37]. Macaques were infected with VN3040 on day 0 and treated with MAb ch61 or control MAbs twice on days 1 and 3 after infection. Body temperatures rose upon infection and decreased after the first injection of MAb ch61, but rose again on days 4–5 (Fig. S2). One of three macaques injected with control MAbs (C3) died on day 4, whereas all three macaques treated with MAb ch61 survived until day 7 after infection (Table 1, Exp. #1). The viral titers in nasal, tracheal, and bronchial samples of macaques treated with MAb ch61 were lower than those of macaques injected with control MAbs after the first injection of MAbs (i.e., on days 2 and 3) (Figs. 3A–C). In one of the MAb ch61-treated macaques (T1), the virus was only slightly detected in the nasal and bronchial samples on days 3–7 (Figs. 3A, C). Although the virus was recovered from the nasal samples of the other treated macaques (T2 and T3), the titers were lower than those of macaques injected with control MAbs (C1, C2, and C3) (Fig. 3A). Infectious viruses were recovered from lungs of most of the macaques even on day 7 (Table 2). Interestingly, the viral titers in nasal, tracheal, and bronchial samples drastically increased after day 4 in one macaque treated with MAb ch61 (T3) (Figs. 3A–C). Similar phenomenon was partially observed in the other treated macaques. Viral titers in tracheal and bronchial samples were often higher in T2 and T3 than in control macaques on days 4–7 (Figs. 3B, C). Accordingly, relatively high titers of the virus was detected in their lungs collected on day 7 (Table 2). Two of the treated macaques (T2 and T3) lost their appetite after virus infection and their clinical scores were increased, but they temporally recovered after injection of MAb ch61 (Fig. 3D). These results indicated that MAb ch61 reduced viral titers in the respiratory secretions of all the treated macaques, although inhibition of viral propagation was temporary in two of the treated macaques. We confirmed that the MAb concentrations on days 3–7 after challenge were maintained at above 20 µg/ml in all treated macaques up to day 7 (Fig. 3E). Thus, to examine the appearance of escape mutants, we sequenced viral RNAs extracted from the tracheal samples collected from MAb ch61-treated macaques on day 5. We found amino acid substitutions identical to those seen in the escape mutants selected in vitro (i.e., K193N or K193E) in 83% (5/6) and 25% (3/12) of the cloned viral genes obtained from T1 and T3, respectively, indicating that viral escape occurred during the treatment period.

**Table 1:** Summary of treatments and survival rates of macaques.

Exp.	Animal ID	CP and CA	MAb	Peramivir	Survival/Total
#1	C1–C3	−	ch113+ch226	−	2/3
	T1–T3	−	ch61	−	3/3
#2	IC1–IC3	+	ch113+ch226	−	0/3
	IT1–IT5	+	ch61	−	3/4^a^
#3	ICP1–ICP3	+	ch113+ch226	+	1/3
	ITP1–ITP3	+	ch61	+	2/3
All^b^	C, IC, ICP	− or +^c^	ch113+ch226	− or +^d^	3/9^e^
	T, IT, ITP	− or +	ch61	− or +	8/10^e^

a One animal (IT3) that died most likely of bacterial infection was excluded.

b Sums of macaques used in three experiments are shown.

c CP and CA for immunosuppression were used in Exp. #2 and #3 but not in #1.

d Peramivir treatments were combined in Exp. #3 but not in #1 and #2.

e Significantly different survival rates between the two groups (chi-square test, p<0.05).

**Table 2:** Virus titers in the lungs of macaques.

		Log_10_ TCID_50_/g^a^					
Animal	Autopsy	RU^b^	RM^b^	RL^b^	LU^b^	LM^b^	LL^b^
C1	Day 7	1.67	2.23	4.00	ND^c^	ND	2.00
C2	Day 7	ND	1.67	ND	2.00	ND	1.67
C3	Day 3	3.33	3.33	4.50	3.50	3.67	4.67
T1	Day 7	ND	ND	ND	ND	ND	ND
T2	Day 7	ND	4.23	6.00	4.50	ND	4.00
T3	Day 7	4.67	5.50	5.50	4.67	3.00	4.83
IC1	Day 3	6.00	7.33	7.67	8.00	6.67	7.50
IC2	Day 5	6.00	5.50	5.67	3.23	4.50	5.67
IC3	Day 4	4.50	2.23	4.50	2.33	3.50	4.67
IT1	Day 7	5.00	4.00	4.67	3.33	2.67	3.33
IT2	Day 7	3.50	3.50	1.83	1.67	2.00	4.23
IT3	Day 6	ND	ND	ND	2.33	ND	ND
IT4	Day 4	ND	ND	ND	2.50	1.67	3.67
IT5	Day 7	ND	3.00	2.67	ND	5.33	ND
ICP1	Day 5	ND	ND	ND	3.00	ND	ND
ICP2	Day 7	ND	ND	ND	ND	ND	ND
ICP3	Day 4	ND	ND	ND	ND	ND	1.67
ITP1	Day 7	ND	ND	ND	ND	ND	ND
ITP2	Day 7	ND	ND	ND	ND	ND	ND
ITP3	Day 3	1.67	ND	ND	ND	ND	ND

a After autopsy on indicated days after virus infection, lung tissue samples were collected.

The lung tissue homogenates were prepared and used to infect MDCK cells to determine TCID_50_ per gram of tissue.

b RU, right upper lobe; RM, right middle lobe; RL, right lower lobe; LU, left upper lobe; RM, right middle lobe; LL, left lower lobe.

c Not detected (detection limit was 10^1.5^ TCID_50_/g).

### Efficacy of anti-H5 antibody treatment in immunocompromised macaques infected with H5N1 HPAI virus isolated from a human patient

To further examine the protective potential of MAb ch61, we used an immunocompromised macaque model with influenza virus infection [29]. Macaques were pretreated with CP and CA and then infected with VN3040 on day 0. Increased body temperature was observed after infection in most of the macaques (IC1, IC2, IC3, IT1, IT2, IT3, and IT5) (Fig. S3). Body temperatures that rose upon infection decreased after the treatment with MAb ch61 in IT1 and IT2, but rose again on days 6–7. All three macaques injected with control MAbs succumbed to infection by day 5 (IC1, IC2, and IC3), whereas two (IT3 and IT4) of the five macaques injected with MAb ch61 also died on days 6 and 4, respectively (Table 1, Exp. #2).

**Figure 4:**
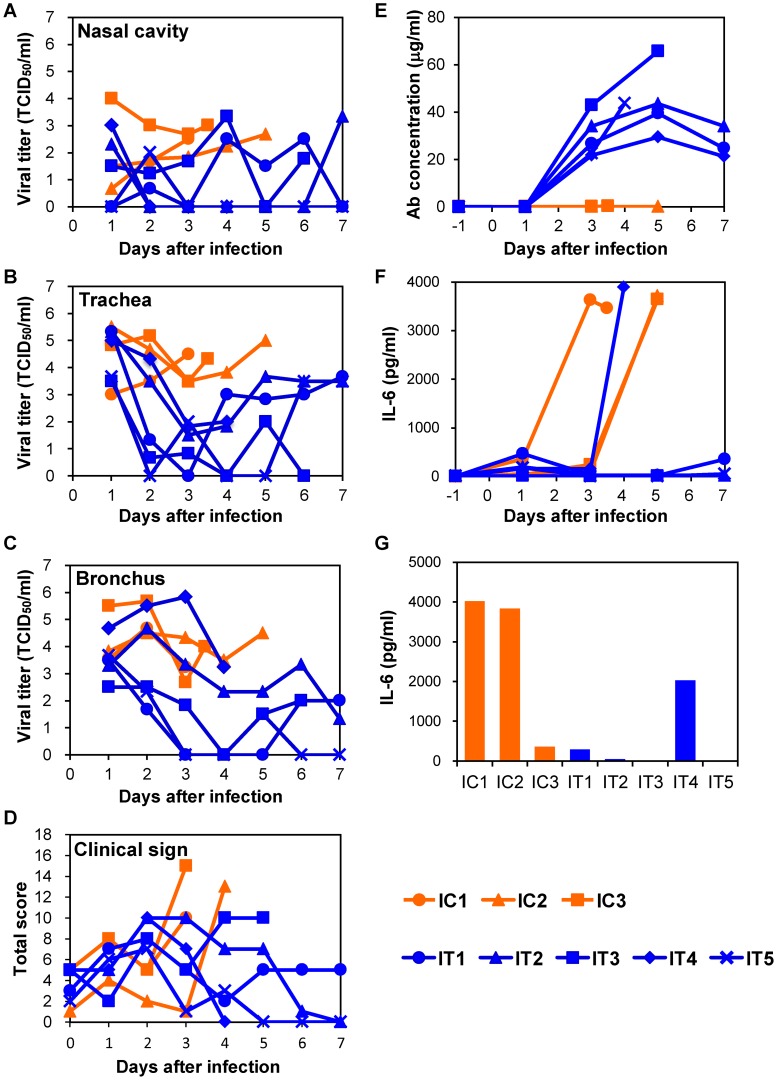
Protection of immunocompromised macaques treated with MAb ch61 from VN3040 infection. Macaques pretreated with CP and CA were infected and injected intravenously with control MAbs (IC1–IC3, orange) and MAb ch61 (IT1–IT5, blue) as described in the legend of Figure 3. Viral titers in nasal (A), tracheal (B), and bronchial (C) swab samples were determined using MDCK cells. Viral titers under the detection limit are indicated as 0. Clinical signs were scored with the parameters shown in Table S1 (D). Concentrations of MAb ch61 (E) and IL-6 (F: serum and G: lung) were examined as described in the legend of Figure 3. Lung tissue samples were collected at autopsy from IC1 on day 3, from IC2 on day 5, from IC3 on day 4, from IT3 on day 6, and from IT4 on day 4.

Infectious viruses were consistently detected in the nasal, tracheal, and bronchial samples of macaques injected with control MAbs until death (Figs. 4A–C). On the other hand, the viral titers in the nasal samples of IT2, IT4, and IT5, and those in the tracheal samples of all five macaques treated with MAb ch61 decreased on days 2 and 3 (i.e., after injection of the antibody) (Figs. 4A, B). It was also noted that the viral titers in the bronchial samples of IT1, IT3, and IT5 were markedly reduced on days 2 and 3 (Fig. 4C). However, in the bronchial samples of IT2 and IT4, the titers on days 2 and 3 were similar to those of control macaques (Fig. 4C). Clinical scores in IT2 and IT5 were improved (clinical score = 0) on day 7 and IT1 slightly regained its appetite after MAb treatment (Fig. 4D). The viral titers increased on days 4–7 in the trachea and bronchial samples of some of the treated macaques (e.g., IT1 and IT2), as was the case with treatment of immunocompetent macaques. Furthermore, infectious viruses were detected in all lobes of their lungs, while the virus replication in the lungs of the other treated macaques was limited on day 7 (Table 2). Viruses with the K193R substitution in HA were recovered from the tracheal samples of IT1 and IT2 (11/11 and 17/18 of the cloned HA genes, respectively), whereas the concentrations of MAb ch61 circulating in the serum on days 3–7 after challenge were maintained at above 20 µg/ml in all treated macaques (Fig. 4E). These results indicated that treatment with MAb ch61 resulted in reduced viral loads and partial protection from lethal HPAI virus infection in immunosuppressed macaques, though this MAb treatment might select escape mutants.

### Efficacy of combination therapy using anti-H5 antibody and a neuraminidase inhibitor in immunocompromised macaques infected with H5N1 HPAI virus isolated from a human patient

**Figure 5:**
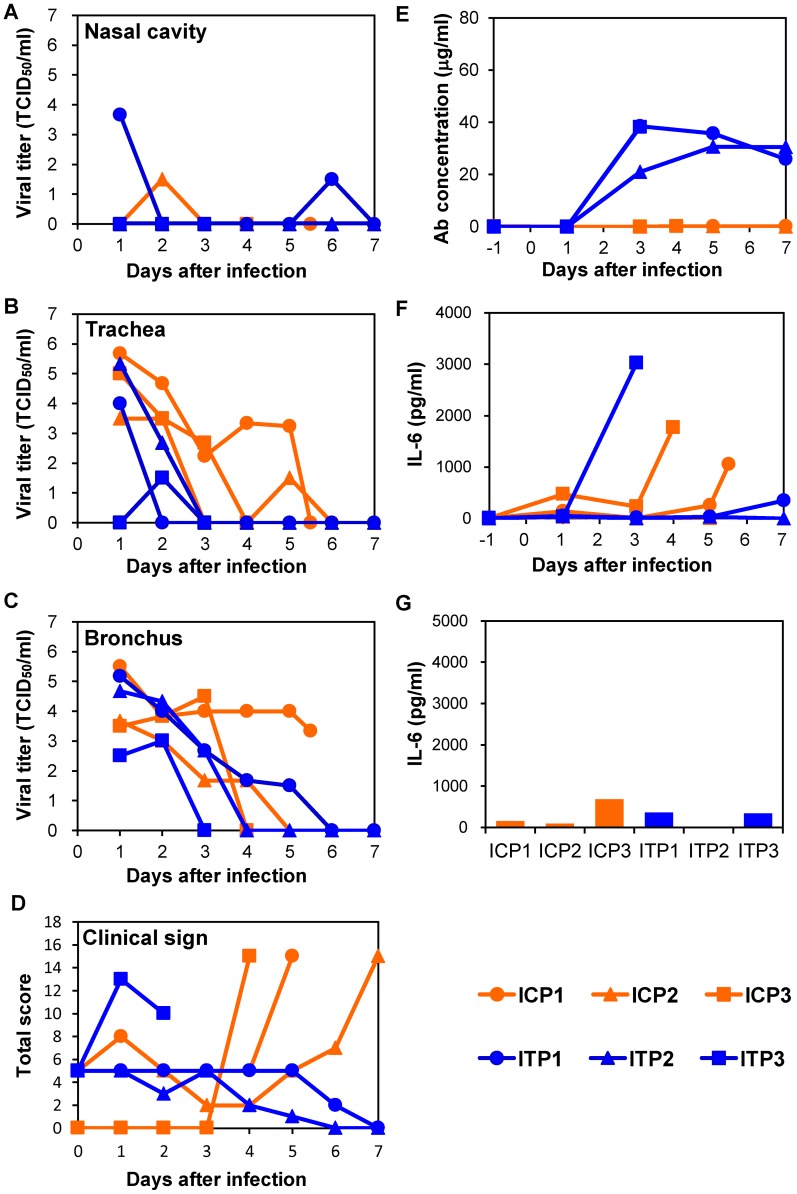
Protection of immunocompromised macaques treated with MAb ch61 and peramivir from VN3040 infection. Macaques pretreated with CP and CA were infected and injected intravenously with control MAbs (ICP1–ICP3, orange) and MAb ch61 (ITP1–ITP3, blue) and with peramivir as described in the legend of Figure 3. Viral titers in nasal (A), tracheal (B), and bronchial (C) swab samples were determined using MDCK cells. Clinical signs were scored with the parameters shown in Table S1 (D). Viral titers under the detection limit are indicated as 0. Concentrations of MAb ch61 (E) and IL-6 (F: serum and G: lung) were examined as described in the legend of Figure 3. Lung tissue samples were collected at autopsy from ITP3 on day 3, from ICP3 on day 4, from ICP1 on day 5, and from other macaques on day 7.

Since escape mutants were frequently selected in macaques treated with MAb ch61 alone, we examined combination therapy with MAb ch61 and the neuraminidase inhibitor peramivir to further reduce viral replication and the emergence of escape mutants. CP- and CA-pretreated macaques were infected with VN3040 and then MAbs were injected on days 1 and 3 in addition to continuous administration of peramivir on days 1–5. Two macaques treated with peramivir alone had to be humanely euthanized on days 5 and 4 (ICP1 and ICP3, respectively), whereas one macaque that received the combined treatment also died on day 3 (ITP3) (Table 1, Exp. #3). Increased body temperature was observed in two control and one ch61-treated macaques (ICP1, ICP3, and ITP2) (Fig. S4). The viral titers in the nasal and tracheal samples of macaques treated with both MAb ch61 and peramivir were almost undetectable after day 3 (Figs. 5A, B). Unlike MAb treatment alone (Figs. 3 and 4), no increase of the viral titer or body temperature was observed on days 4–7 in surviving macaques treated with MAb ch61 together with peramivir (Figs. 5A–C and Fig. S4) and the concentrations of MAb ch61 in the serum were maintained at above 20 µg/ml on days 3–7 after challenge in these macaques (Fig. 5E). Accordingly, infectious viruses were only slightly detected in the limited parts of lungs of the macaques (Table 2) and escape mutations (i.e., K193N or K193E) were not found in the cloned viral genes (0/11) obtained from the MAb ch61-treated macaques. Along with the reduced viral recovery from the samples, clinical scores in ITP1 and ITP2 were generally improved on day 7. These results indicated that combination therapy with MAb ch61 and peramivir inhibited viral propagation more efficiently than MAb or peramivir treatment alone, which might also result in reduced selection of escape mutants and improved survival after H5N1 HPAI virus infection in macaques.

### Correlation between disease severity and elevated levels of IL-6

To determine the cause of death of the macaques, we examined inflammation by measuring IL-6 production in sera and lung tissues. In the immunocompetent macaque model, an elevated IL-6 level was observed on day 3 in the serum of one macaque (C3) that died on day 4 but not in the other macaques (Fig. 3F). In addition, the lung IL-6 level of C3 on day 3 was markedly higher than those of the other macaques (Fig. 3G). In the immunocompromised macaque model, a marked increase of IL-6 was detected in the sera and/or lung tissues of all macaques injected with control MAbs (Figs. 4F, G). Similarly, increased IL-6 levels were detected in IT4. In a MAb ch61-treated macaque that died on day 6 (IT3), bacterial infection was detected in the cerebral ventricle (data not shown) and the rapid IL-6 response was not observed, suggesting that this macaque died of bacterial meningitis, not virus infection. In the combination therapy experiment, increased levels of IL-6 were detected in the sera of ICP1, ICP3, and ITP3, all of which were humanely euthanized or died after infection (Fig. 5F). IL-6 levels in lung tissues were relatively high in ICP3 and ITP3 (Fig. 5G). Consistent with some human cases previously described [38], [39], these results suggested that increases of IL-6 in the serum and lungs might be associated with systemic inflammatory responses leading to death. While increased production of TNF-α and IL-1β were also seen in the macaques with severe disease, the other cytokines tested were unlikely correlated with disease severity of the macaques (Figs. S5, S6). Since elevated levels of IL-6, TNF-α and IL-1β are likely involved in a variety of systemic inflammatory states that are associated with endothelial barrier dysfunction, these cytokines could be important mediators of increased endothelial permeability, which might result in systemic organ failure caused by H5N1 HPAI virus infection.

### Gross pathology and histopathology of macaques infected with H5N1 HPAI virus

**Figure 6:**
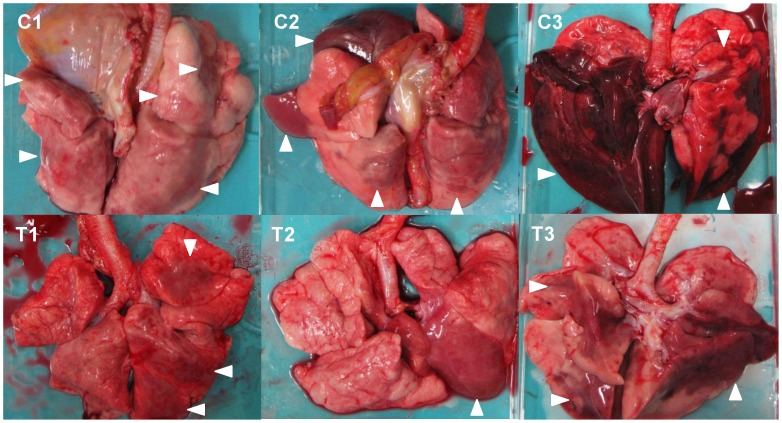
Gross pathological changes of the lungs of immunocompetent macaques infected with VN3040. Macaques were treated with control antibodies (C1–C3) or MAb ch61 (T1–T3). Macaque C3 was autopsied 3 days after virus infection. The other macaques were autopsied 7 days after virus infection. Dark red lesions indicated by white arrowheads show macroscopic inflammation, hemorrhage, and congestion.

**Figure 7:**
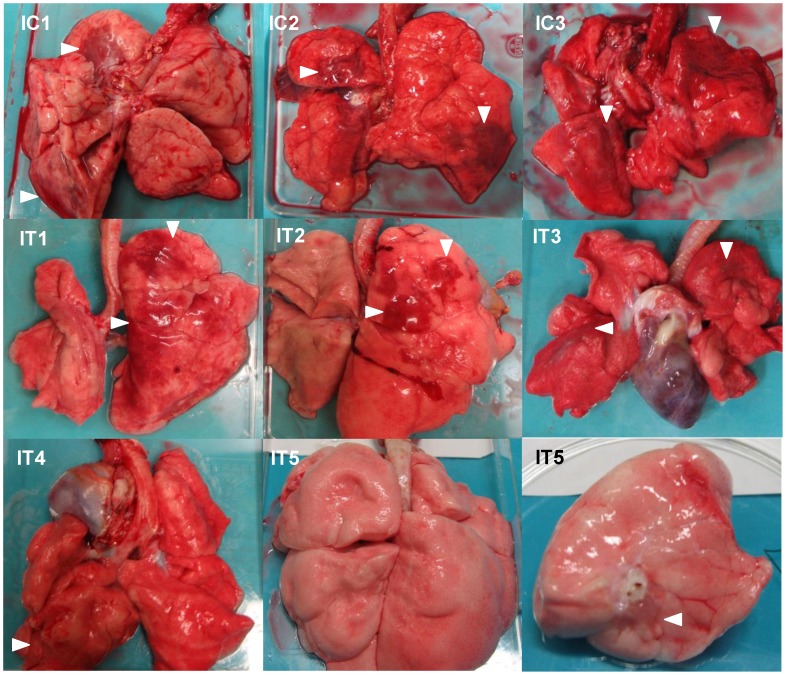
Gross pathological changes of the lungs of immunosuppressed macaques infected with VN3040. Immunosuppressed macaques were treated with control antibodies (IC1–IC3) or MAb ch61 (IT1–IT5). Macaques IC1, IC2, and IC3 were autopsied 3 days, 5 days, and 4 days after virus infection, respectively. Macaques IT3 and IT4 were autopsied 6 days and 4 days after virus infection, respectively. Macaques IT1, IT2, and IT5 survived during the observation period and were autopsied 7 days after virus infection. The upper lobe of the left lung of IT5 is shown in the lower right picture. Dark red lesions indicated by white arrowheads show macroscopic inflammation, hemorrhage, and congestion.

**Figure 8:**
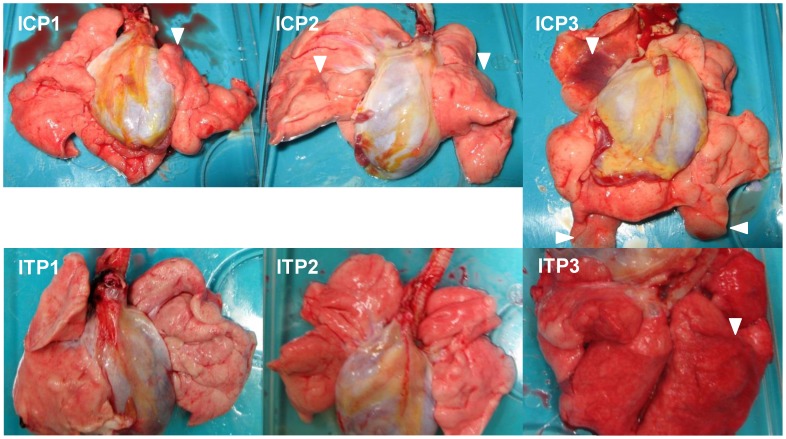
Gross pathological changes of the lungs of immunosuppressed and peramivir-treated macaques infected with VN3040. Immunosuppressed macaques were treated with control antibodies (ICP1–ICP3) or MAb ch61 (ITP1–ITP3) together with peramivir. Macaques ICP1, ICP3, and ITP3 were autopsied 5 days, 4 days, and 3 days after virus infection, respectively. The other macaques survived during the observation period and were autopsied 7 days after virus infection. Dark red lesions indicated by white arrowheads show macroscopic congestion.

To evaluate the progression of disease after the antibody treatment, we examined the lung pathology of the macaques subjected to autopsy. Macroscopically, dark red areas representing inflammation and congestion were larger in the lungs of control immunocompetent macaques (C1–C3) than in the lungs of two immunocompetent macaques treated with MAb ch61 (T1 and T2) (Fig. 6). The dark red area was larger in the lung of T3 than in the lungs of T1 and T2. These findings were concordant with virus titers in the lungs collected at autopsy (Table 2). In immunosuppressed macaques treated with control antibodies (IC1–IC3), the macroscopic lesions with inflammation, hemorrhage, and congestion in the lungs were much smaller than those in the lungs of immunocompetent macaques (C1–C3) (Figs. 6, 7). In immunosuppressed macaques treated with MAb ch61 (IT1–IT5), the reddish lesions were smaller than those in control macaques (IC1–IC3) (Fig. 7). In particular, macroscopic inflammation in IT5 was observed only around the central bronchus. In immunosuppressed macaques also treated with peramivir, macroscopic reddish lesions were smaller than those in immunocompetent and immunosuppressed macaques treated without peramivir (Figs. 6–8). The lung of ICP3, which died on day 4 after virus infection, had dark red, edematous lesions.

**Figure 9:**
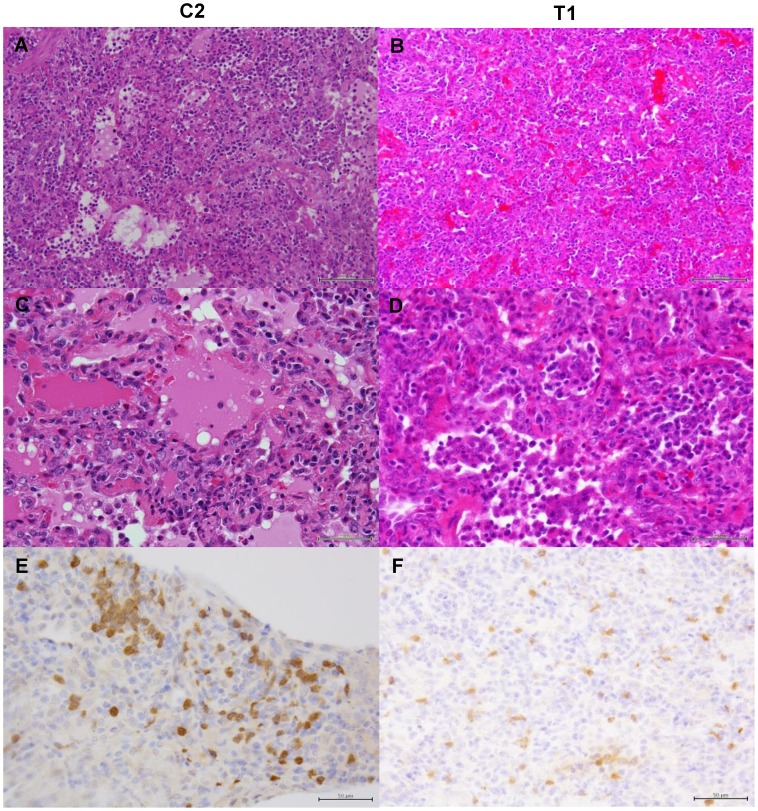
Histological analysis of pneumonia and distribution of viral antigens in immunocompetent macaques infected with VN3040. Tissues were collected from macaques injected with control antibodies (C2) (A, C, E) or MAb ch61 (T1) (B, D, F) on day 7 after virus infection. A, B: low magnification of hematoxylin and eosin (H & E) staining (bar: 100 µm). C, D: high magnification of H & E staining (bar: 50 µm). E, F: sections stained with anti-NP serum (bar: 50 µm).

**Figure 10:**
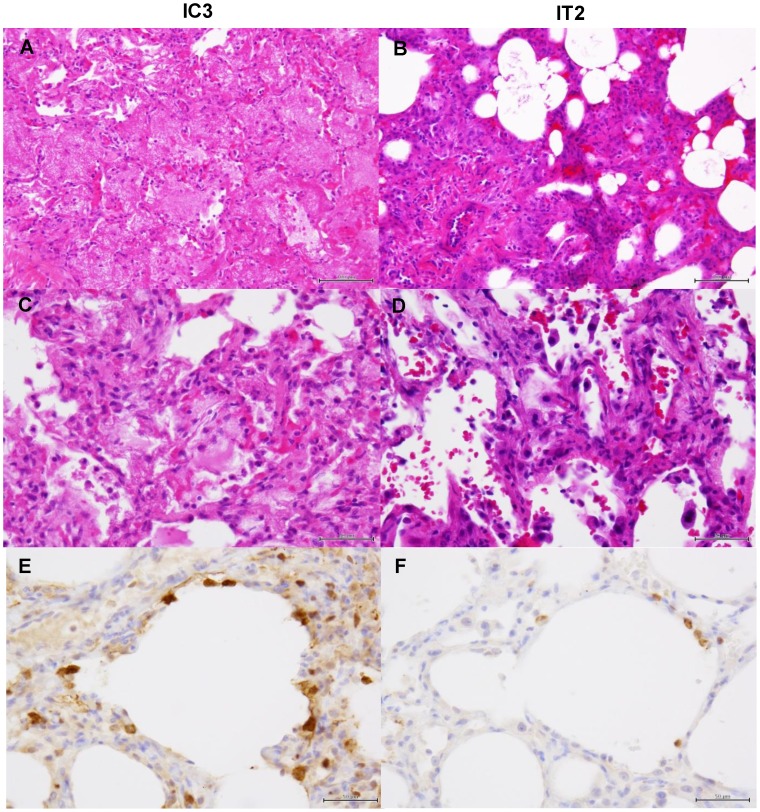
Histological analysis of pneumonia and distribution of viral antigens in immunosuppressed macaques infected with VN3040. Tissues were collected from macaques injected with control antibodies (IC3) (A, C, E) or MAb ch61 (IT2) (B, D, F) on days 4 and 7 after virus infection, respectively. Other details are the same as the legend of Figure 9.

**Figure 11:**
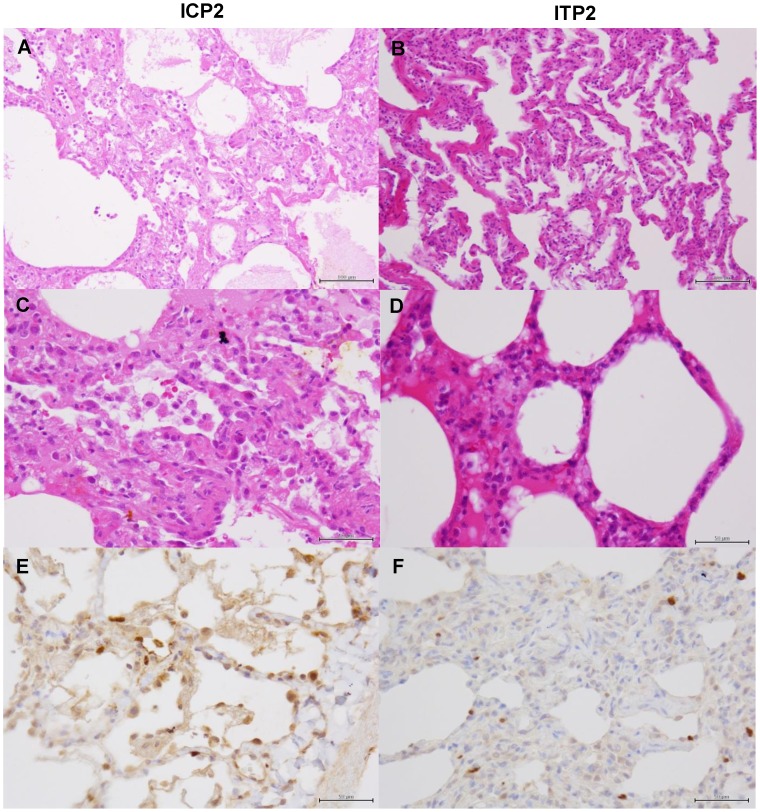
Histological analysis of pneumonia and distribution of viral antigens in immunosuppressed and peramivir-treated macaques infected with VN3040. Tissues were collected from macaques treated with peramivir and injected with control antibodies (ICP2) (A, C, E) or MAb ch61 (ITP2) (B, D, F) on day 7 after virus infection. Other details are the same as the legend of Figure 9.

We then examined histological changes of the lungs collected from the infected macaques (Figs. 9–11). Severe pneumonia reducing air space was seen in a control immunocompetent macaque (C2) and a macaque treated with MAb ch61 (T1) (Figs. 9A, B). In high magnification images, lymphoid and neutrophilic infiltration, thickened alveolar walls, and alveolar edema were observed (Figs. 9C, D). The other macaques (C2, T2, and T3) euthanized on day 7 showed similar histological changes (data not shown). In immunohistochemical staining for the influenza virus antigens, NP-positive cells were widely distributed and accumulated focally in the lung of the control macaque 7 days after virus infection (Fig. 9E). By contrast, NP-positive cells were seen but did not accumulate in the MAb ch61-treated macaque (Fig. 9F). Reduced numbers of NP-positive cells were also seen in the lungs of the other treated macaques (T2 and T3) (data not shown).

In macaques under the immunosuppressed condition, lymphoid infiltration was very mild compared with immunocompetent macaques. In the lung tissue obtained from a control macaque (IC3) at 4 days after virus infection, pulmonary edema was seen in the alveoli, resulting in loss of air space (Figs. 10A, C). In a macaque treated with MAb ch61 (IT2), the air space was decreased and alveolar septa were thickened in part, but the air content was still preserved (Figs. 10B, D). NP-positive cells were seen in the alveolar epithelium of the control macaque more frequently than in that of the MAb ch61-treated macaque (Figs. 10E, F). The cuboidal epithelial cells positive for the NP antigen were type II alveolar epithelial cells. Less severe histological changes and virus infection were also seen in the other treated macaques (IT1 and IT3) compared with the control macaques (data not shown). These differences in the histological changes and frequencies of NP-positive cells between control and MAb ch61-treated macaques were also seen in the macaques treated together with peramivir (Fig. 11).

## Discussion

Current strategies for the control of influenza include vaccination and antiviral drug treatment. Neuraminidase inhibitors have been used for H5N1 HPAI virus infection in humans as well as seasonal influenza caused by viruses of the H1 and H3 HA subtypes. However, the efficacy of the neuraminidase inhibitors on the human H5N1 infections is unclear due to the inevitable lack of adequate control studies. Moreover, drug-resistant H5N1 viruses were indeed detected in patients [40], [41] and, importantly, H5N1 viruses with reduced sensitivity to neuraminidase inhibitors were also isolated from chickens in the endemic area [42]. Thus, alternative strategies for prophylaxis and treatment need to be developed for pandemic preparedness against the H5N1 influenza virus.

Passive transfer of neutralizing antibodies may provide an alternative strategy for both prophylaxis and treatment of pandemic influenza. It was reported that an H5N1 HPAI virus-infected patient recovered after treatment with convalescent plasma, suggesting that passive immunotherapy may be a promising option for the treatment of H5N1 HPAI virus infection [43]. The efficacy of mouse MAbs specific for H5 HAs been evaluated in a mouse model with promising results for both treatments and prophylaxis [44], [45]. However, for clinical use, induction of anti-mouse MAb-specific antibody responses should reduce the neutralizing capacity of given MAbs and also limit the repeated use of mouse antibodies. Thus, passive immunotherapy with human or humanized MAbs has also been tested in mouse and ferret models [46]–[49]. Nevertheless, the protective potential of anti-H5 MAbs remained to be elucidated in a nonhuman primate model of H5N1 HPAI virus infection.

To help develop a clinical antibody therapy, we also generated a human-mouse chimeric monoclonal antibody (MAb ch61) that showed strong neutralizing activity against H5N1 HPAI viruses isolated from humans and evaluated its protective potential in animal models. In particular, we used a cynomolgus macaque model, which simulates the H5N1 HPAI virus infection of humans more faithfully and thus has been used as an animal model for vaccine and pathogenesis studies on influenza virus infection [50]. We found that treatment with MAb ch61 reduced viral loads and partially protected macaques from lethal infection with the H5N1 HPAI virus. It was noteworthy that the protective effect was more prominent in immunosuppressed macaques, which might provide a model of protection against severe clinical disease in immunocompromised patients. Thus, this proof of concept study provides the first evidence that antibody therapy may have beneficial effects in clinical cases of H5N1 HPAI virus infection in humans. Importantly, however, mutant viruses escaping from neutralization by MAb ch61 were recovered from some of the macaques treated with MAb ch61 alone and became predominant by 7 days after infection, whereas reduced virus replication upon treatment with MAb ch61 was observed in most of the treated macaques during the initial phase of infection. These results suggest that, as was shown in a mouse model of H5N1 HPAI virus infection [51], combination therapy using two different MAbs might be needed to prevent the generation of escape mutants and would likely be more beneficial.

Taken together, the results obtained in the present study demonstrated that the therapeutic use of anti-H5 neutralizing MAb ch61 resulted in reduced viral loads and improved protection in a nonhuman primate model of lethal H5N1 virus infection. In addition, it was also shown that combination therapy with the antiviral drug provided better protection and reduced the emergence of escape mutants. Combination therapy with other antibodies recognizing different epitopes may also attenuate symptoms and prevent the selection of escape mutants.

## Supporting Information

Figure S1 **The number of white blood cells in macaques treated with immunosuppressive agents.** The macaques were administered CP intravenously on days −7, −5, −3, −1 and 0 and CA intragastrically from day −7 to day 6 (A, B). A control group was administered saline intravenously and intragastrically (C). The macaques were injected intravenously with control MAbs (orange) and MAb ch61 (blue). Macaques in (C) were injected with peramivir intravenously from day 1 to day 5 in addition to MAbs. Blood was collected on the indicated days. The number of white blood cells (WBC) was counted with a microscope and hemocytometer.
(TIFF)

Figure S2 **Body temperatures of immunocompetent macaques treated with MAbs after infection with VN3040.** Macaques were infected with VN3040 (3×10^6^ PFU) on day 0. The macaques were injected intravenously with control MAbs (C1–C3, orange) or anti-H5 MAb ch61 (T1–T3, blue) on days 1 and 3. Depression of temperature was induced once a day by anesthesia.
(TIFF)

Figure S3 **Body temperatures of immunocompromised macaques treated with MAbs after infection with VN3040.** Macaques were pretreated with CP intravenously and with CA intragastrically. Thereafter, they were infected with VN3040 (3×10^6^ PFU) on day 0. The macaques were injected intravenously with control MAbs (IC1–IC3, orange) or anti-H5 MAb ch61 (IT1–IT5, blue) on days 1 and 3. Depression of temperature was induced once a day by anesthesia.
(TIFF)

Figure S4 **Body temperatures of immunocompromised macaques treated with MAbs and peramivir after infection with VN3040.** Macaques were pretreated with CP intravenously and with CA intragastrically. Thereafter, they were infected with VN3040 (3×10^6^ PFU) on day 0. The macaques were injected intravenously with control MAbs (ICP1–ICP3, orange) or anti-H5 MAb ch61 (ITP1–ITP3, blue) on days 1 and 3, and with peramivir on days 1 to 5. Depression of temperature once a day was induced by anesthesia.
(TIFF)

Figure S5 **Cytokine patterns in the sera of macaques after infection with VN3040.** Cytokine concentrations in the serum samples were measured as described in the Materials and Methods section. Left column: immunocompetent macaques (Exp. #1), middle column: immunosuppressed macaques (Exp. #2), right column: immunosuppressed macaques treated with peramivir (Exp. #3).
(PDF)

Figure S6 **Cytokine patterns in the lungs of macaques after infection with VN3040.** Cytokine concentrations in the lung tissue homogenates were measured as described in the Materials and Methods section. Left column: immunocompetent macaques (Exp. #1), middle column: immunosuppressed macaques (Exp. #2), right column: immunosuppressed macaques treated with peramivir (Exp. #3).
(PDF)

Table S1 **Clinical scoring used in this study.** Animals were monitored during the study to be clinically scored.
(DOCX)
