# Supplementary figures and images for: Protective Efficacy of Passive Immunization with Monoclonal Antibodies in Animal Models of H5N1 Highly Pathogenic Avian Influenza Virus Infection
Source: PLoS Pathog. 2014 Jun 12;10(6):e1004192. doi: 10.1371/journal.ppat.1004192 (PMC4055766; doi:10.1371/journal.ppat.1004192)

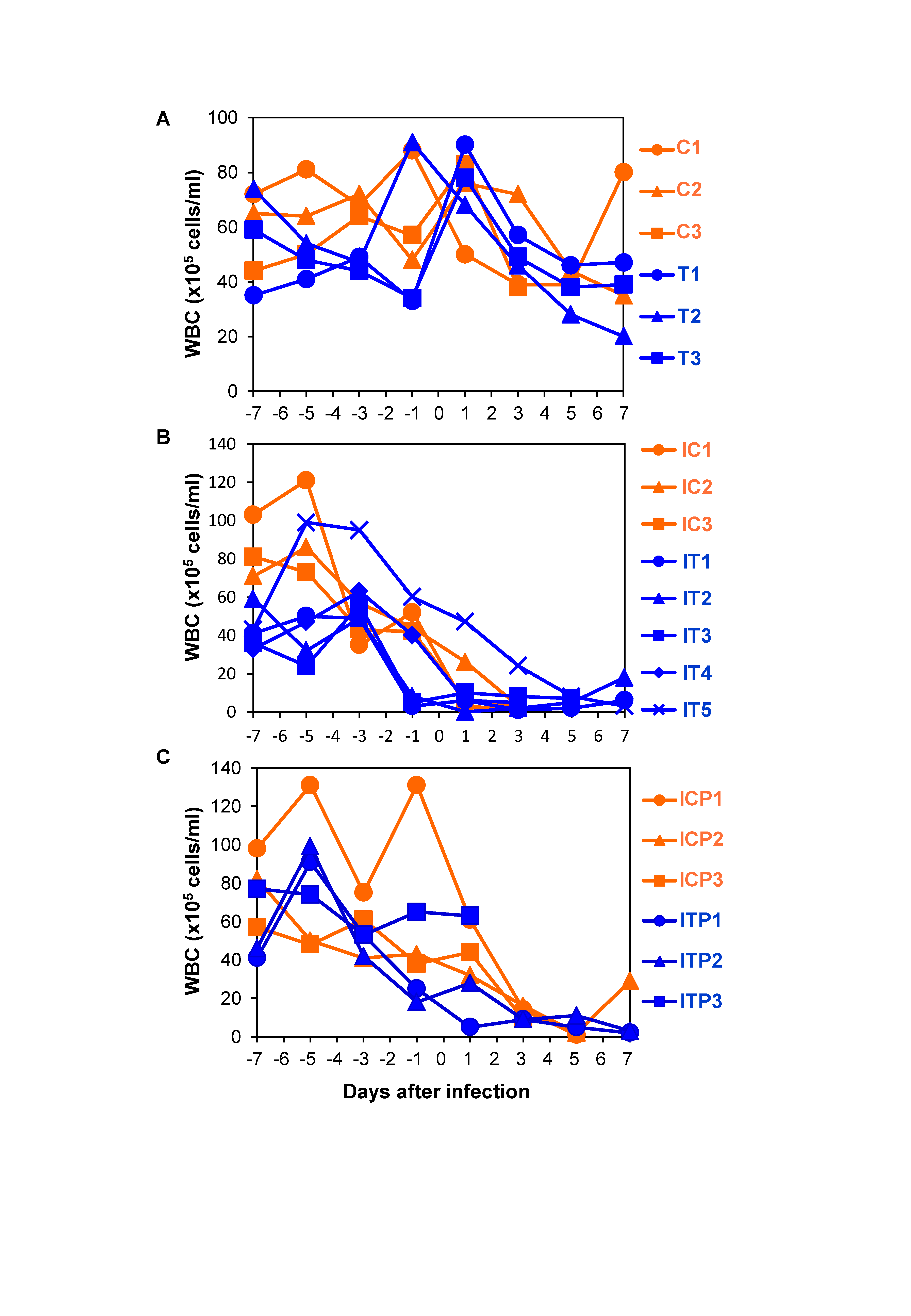

Supplement: Figure S1 — The number of white blood cells in macaques treated with immunosuppressive agents. The macaques were administered CP intravenously on days −7, −5, −3, −1 and 0 and CA intragastrically from day −7 to day 6 (A, B). A control group was administered saline intravenously and intragastrically (C). The macaques were injected intravenously with control MAbs (orange) and MAb ch61 (blue). Macaques in (C) were injected with peramivir intravenously from day 1 to day 5 in addition to MAbs. Blood was collected on the indicated days. The number of white blood cells (WBC) was counted with a microscope and hemocytometer. (TIFF) [file ppat.1004192.s001.tiff]

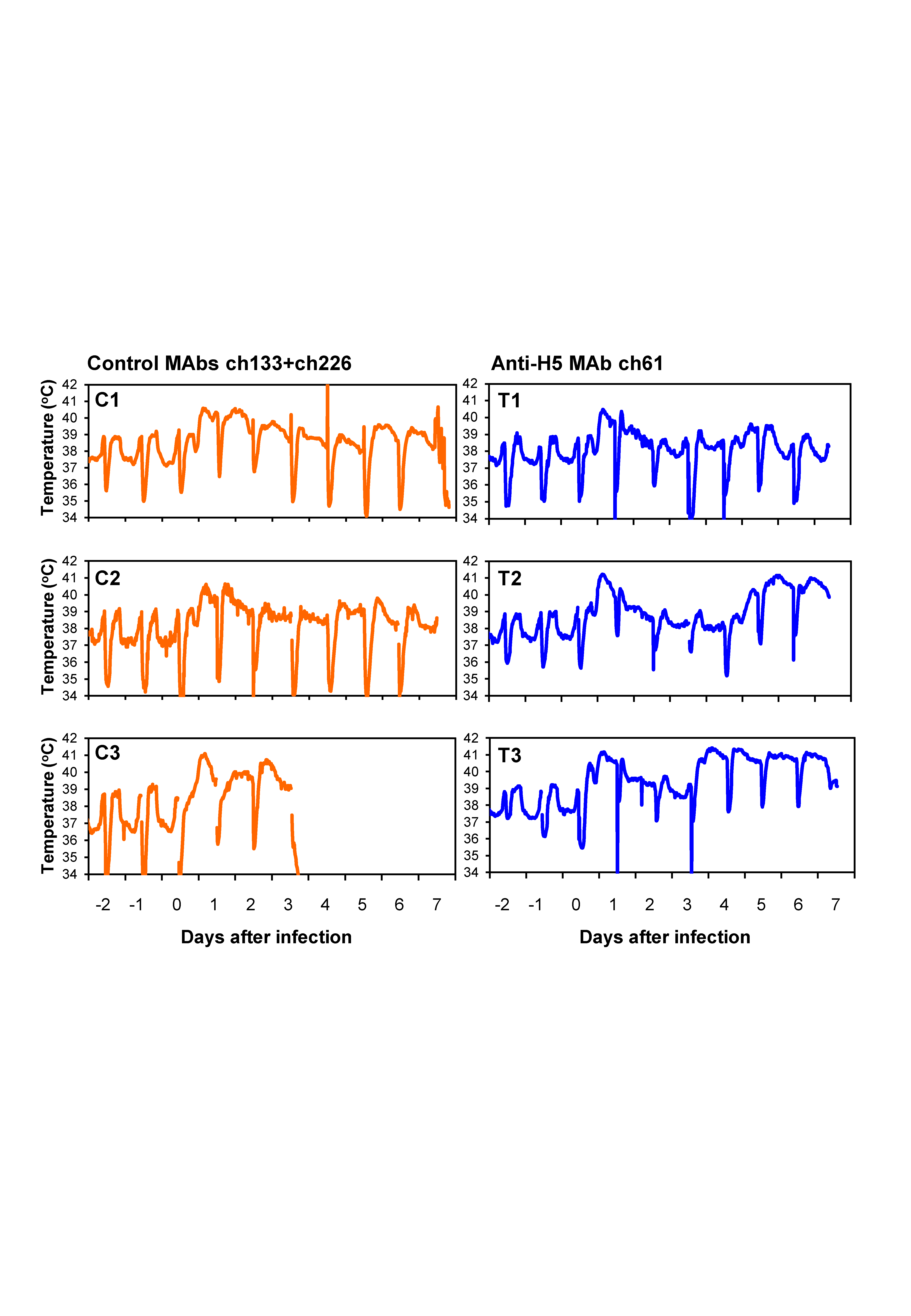

Supplement: Figure S2 — Body temperatures of immunocompetent macaques treated with MAbs after infection with VN3040. Macaques were infected with VN3040 (3×10^6^ PFU) on day 0. The macaques were injected intravenously with control MAbs (C1–C3, orange) or anti-H5 MAb ch61 (T1–T3, blue) on days 1 and 3. Depression of temperature was induced once a day by anesthesia. (TIFF) [file ppat.1004192.s002.tiff]

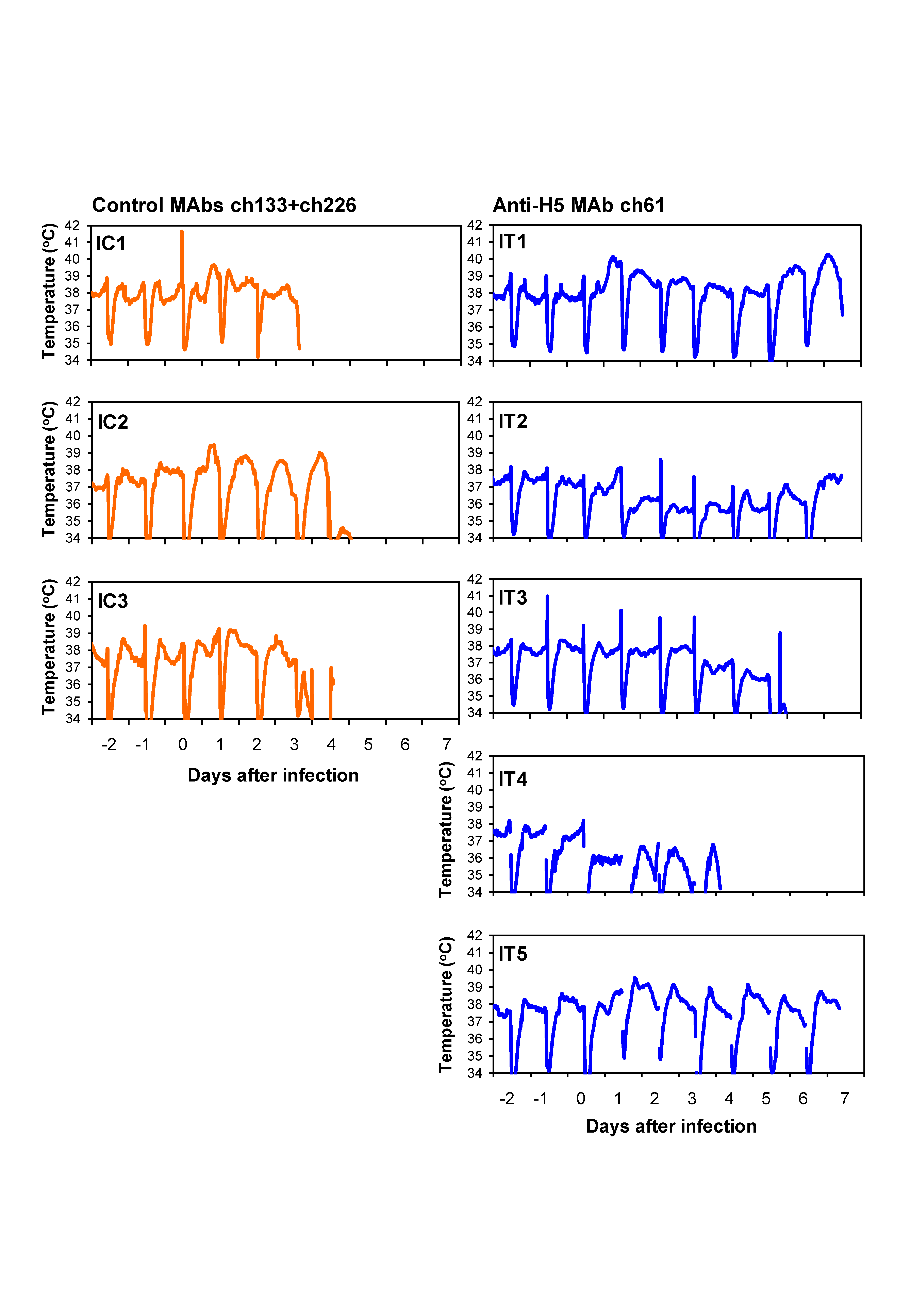

Supplement: Figure S3 — Body temperatures of immunocompromised macaques treated with MAbs after infection with VN3040. Macaques were pretreated with CP intravenously and with CA intragastrically. Thereafter, they were infected with VN3040 (3×10^6^ PFU) on day 0. The macaques were injected intravenously with control MAbs (IC1–IC3, orange) or anti-H5 MAb ch61 (IT1–IT5, blue) on days 1 and 3. Depression of temperature was induced once a day by anesthesia. (TIFF) [file ppat.1004192.s003.tiff]

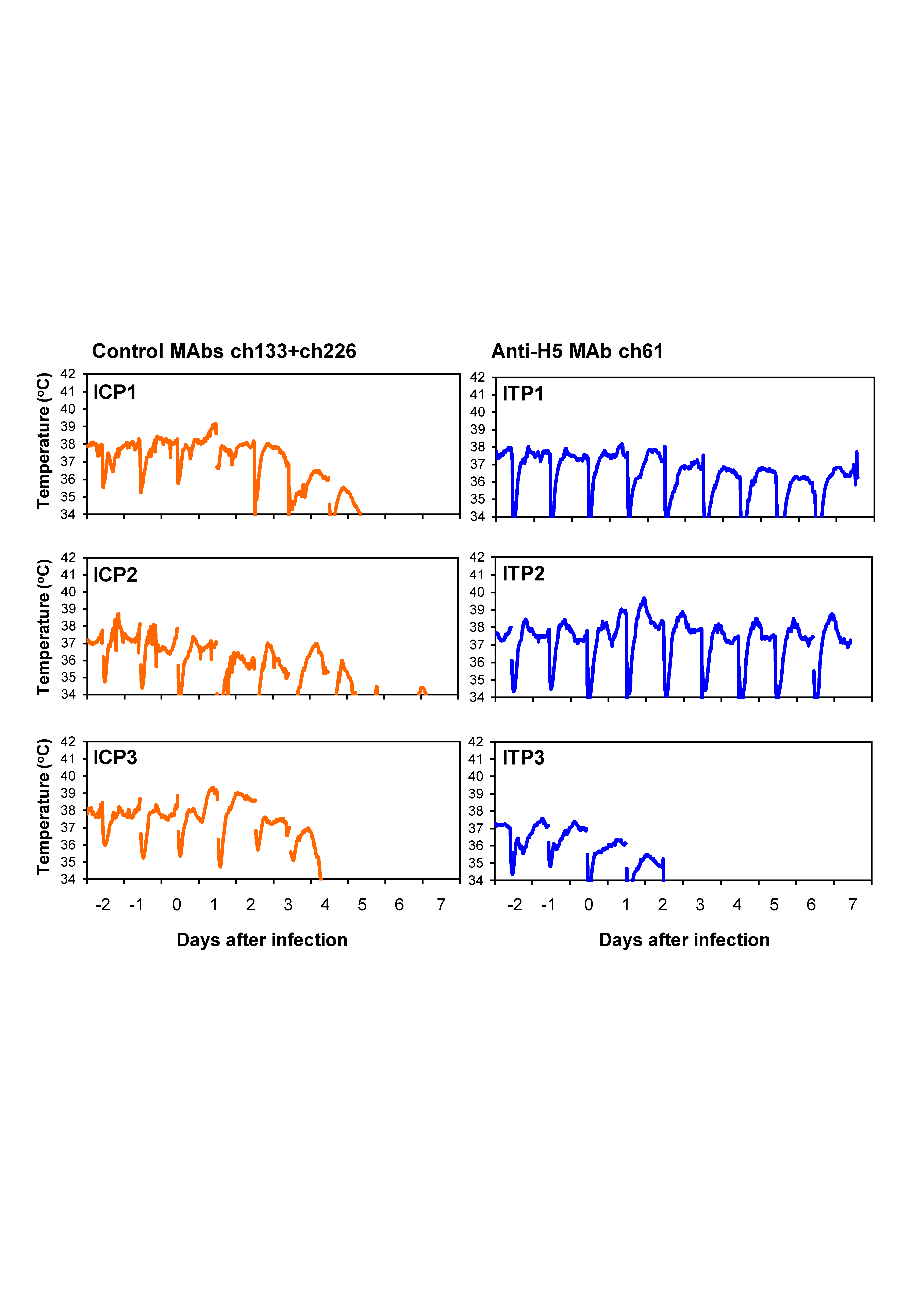

Supplement: Figure S4 — Body temperatures of immunocompromised macaques treated with MAbs and peramivir after infection with VN3040. Macaques were pretreated with CP intravenously and with CA intragastrically. Thereafter, they were infected with VN3040 (3×10^6^ PFU) on day 0. The macaques were injected intravenously with control MAbs (ICP1–ICP3, orange) or anti-H5 MAb ch61 (ITP1–ITP3, blue) on days 1 and 3, and with peramivir on days 1 to 5. Depression of temperature once a day was induced by anesthesia. (TIFF) [file ppat.1004192.s004.tiff]

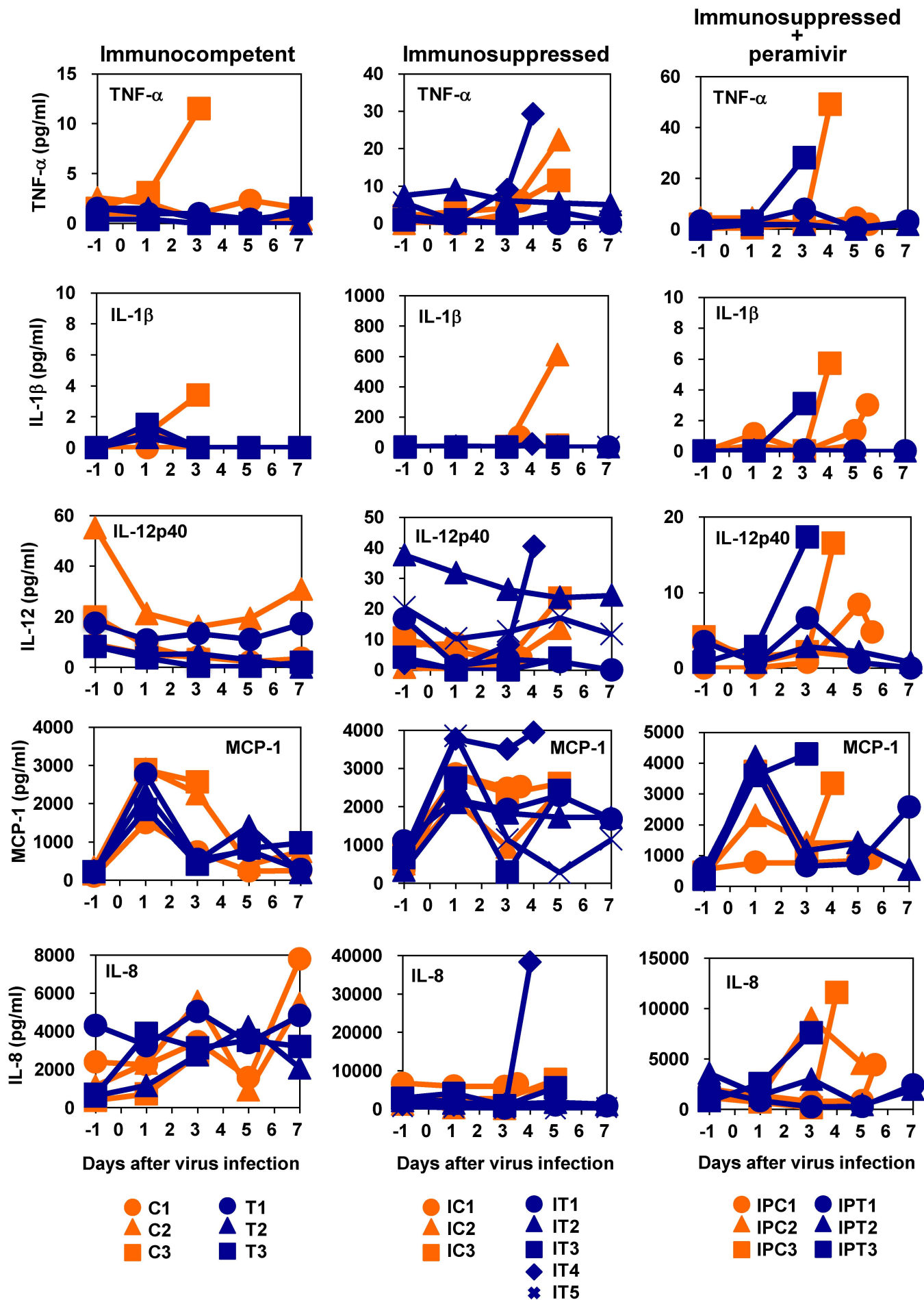

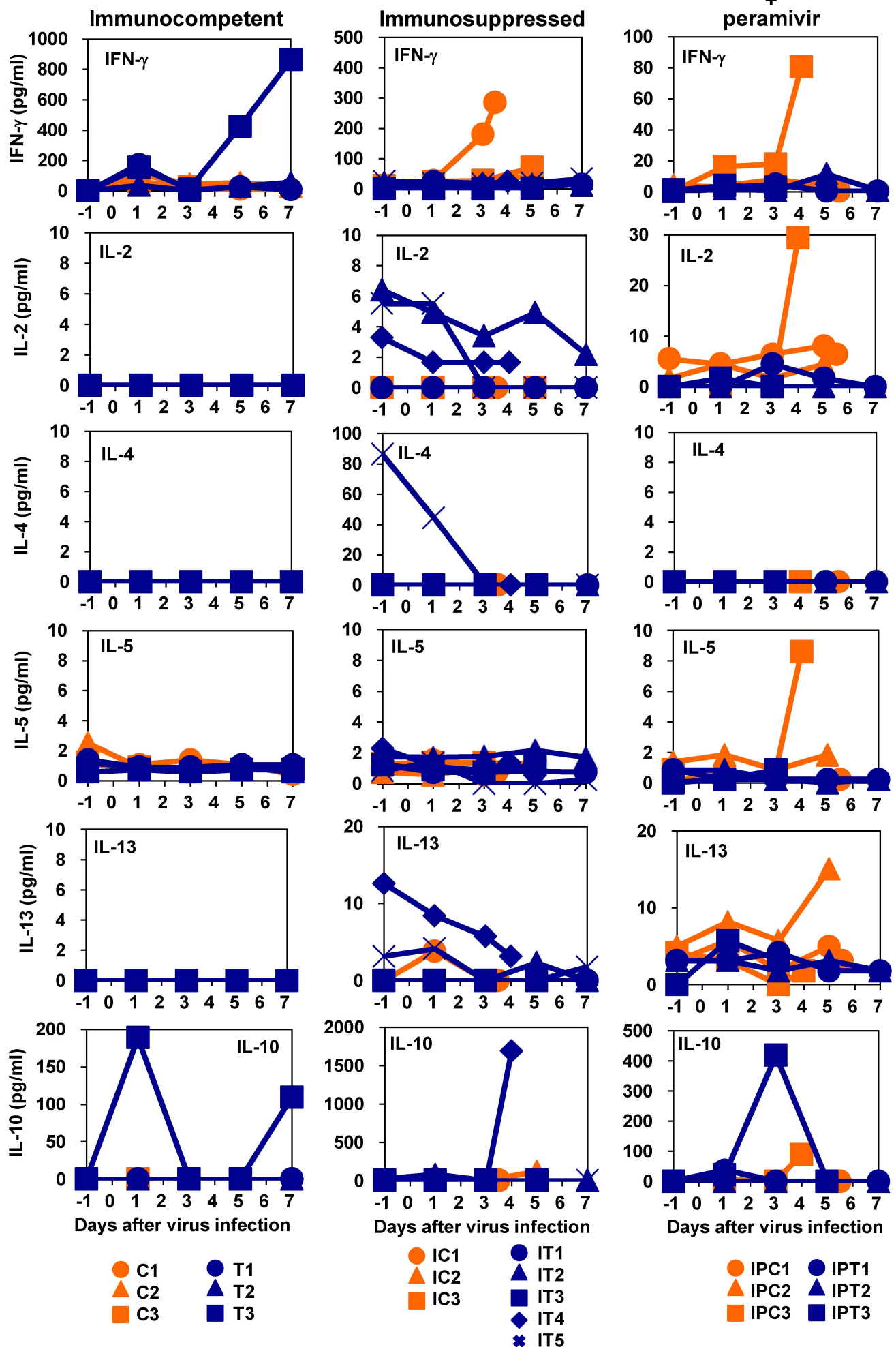

Supplement: Figure S5 — Cytokine patterns in the sera of macaques after infection with VN3040. Cytokine concentrations in the serum samples were measured as described in the Materials and Methods section. Left column: immunocompetent macaques (Exp. #1), middle column: immunosuppressed macaques (Exp. #2), right column: immunosuppressed macaques treated with peramivir (Exp. #3). (PDF) [file ppat.1004192.s005.pdf]

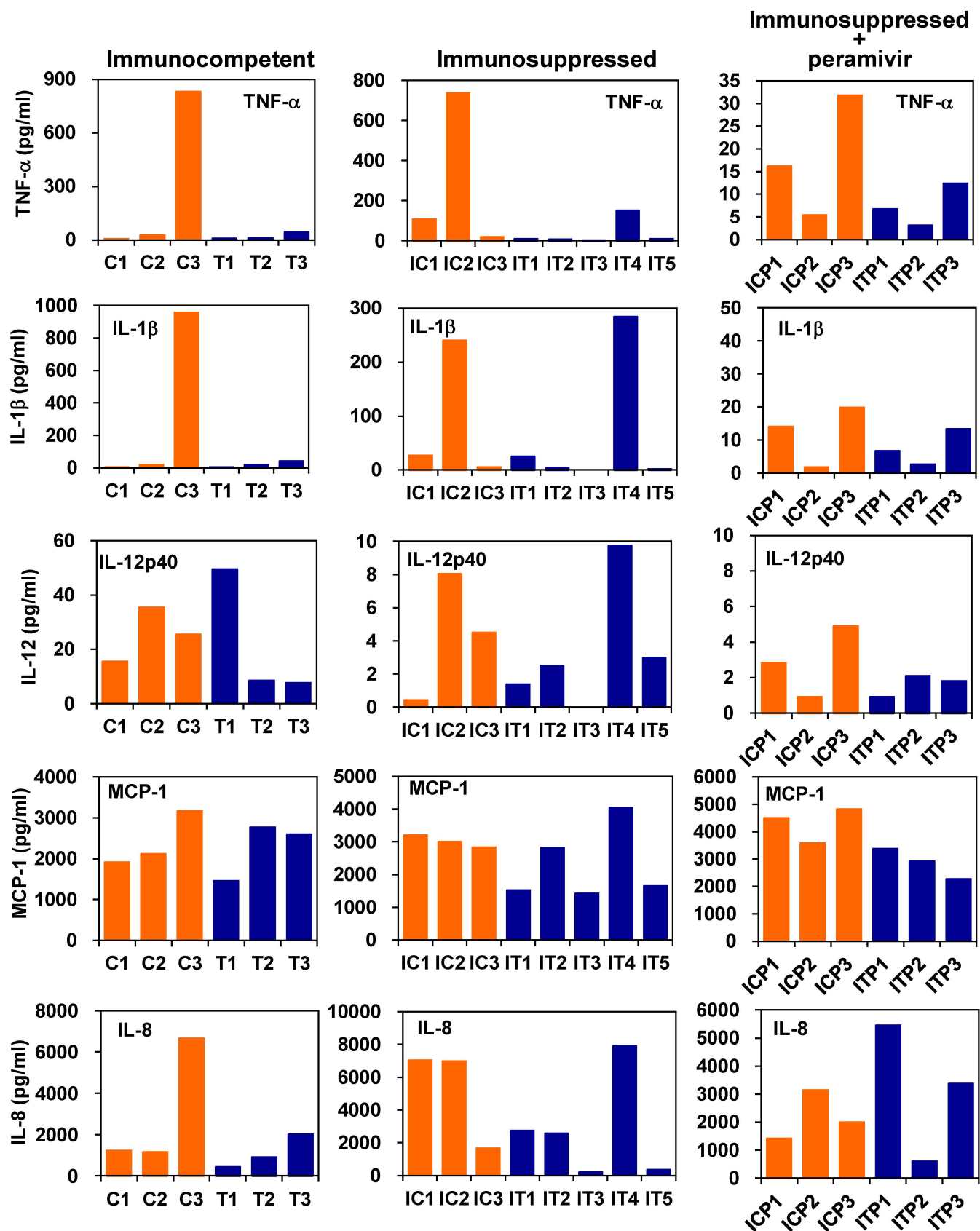

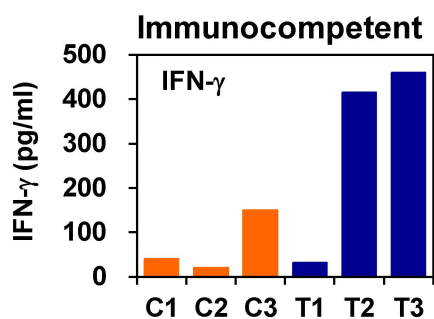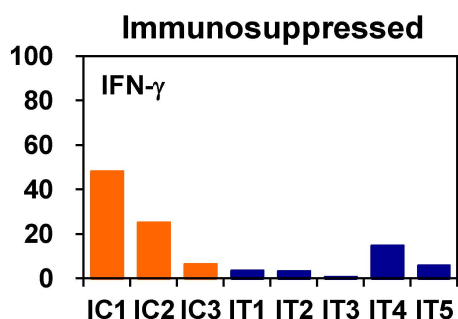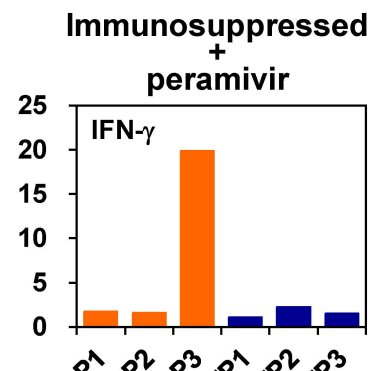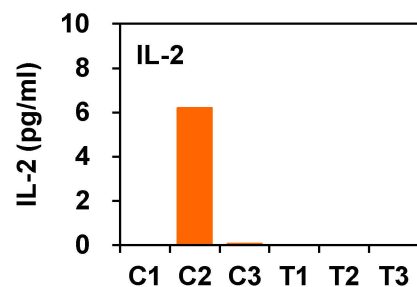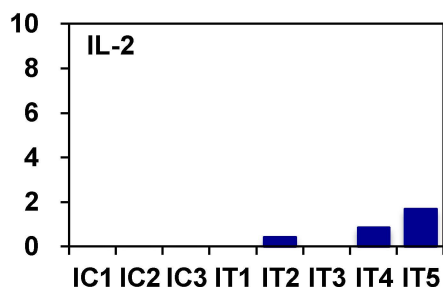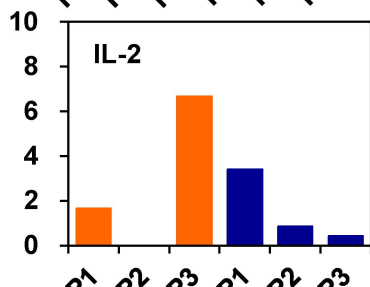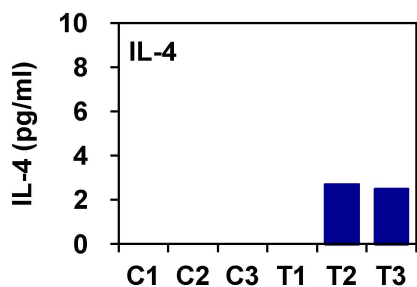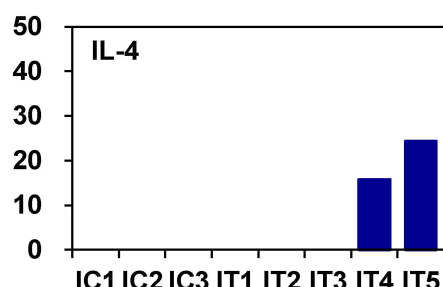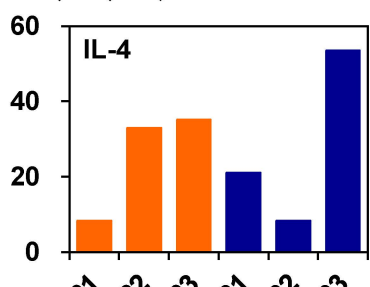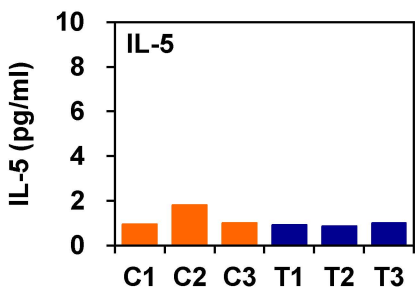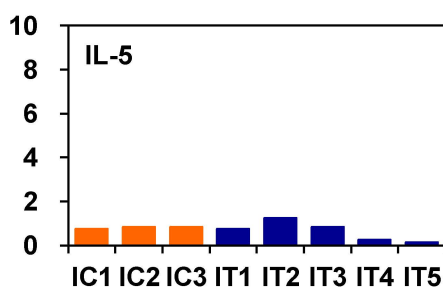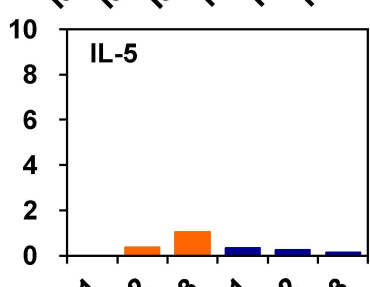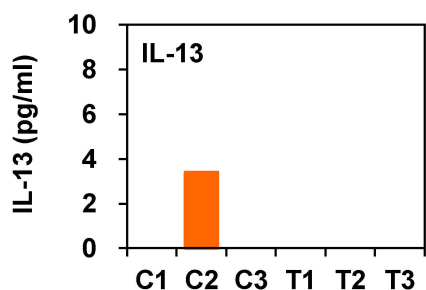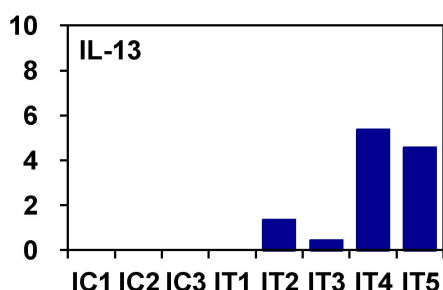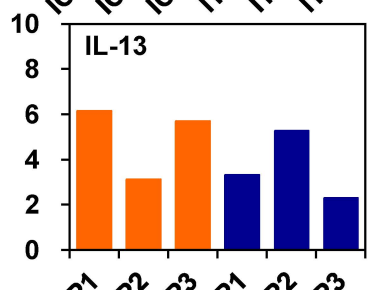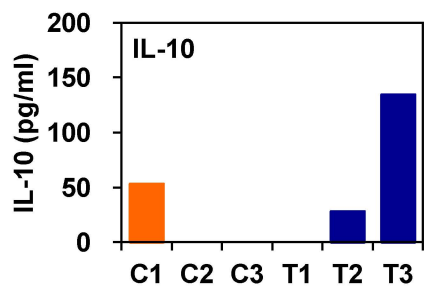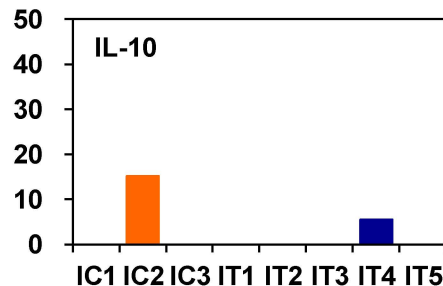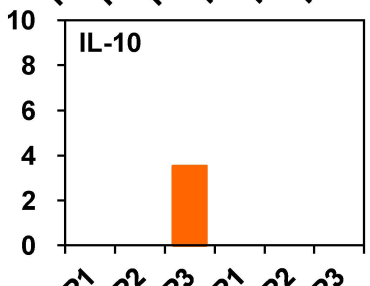

Supplement: Figure S6 — Cytokine patterns in the lungs of macaques after infection with VN3040. Cytokine concentrations in the lung tissue homogenates were measured as described in the Materials and Methods section. Left column: immunocompetent macaques (Exp. #1), middle column: immunosuppressed macaques (Exp. #2), right column: immunosuppressed macaques treated with peramivir (Exp. #3). (PDF) [file ppat.1004192.s006.pdf]
