# Supplementary material for: Protective Efficacy of Passive Immunization with Monoclonal Antibodies in Animal Models of H5N1 Highly Pathogenic Avian Influenza Virus Infection
Source: PLoS Pathog. 2014 Jun 12;10(6):e1004192. doi: 10.1371/journal.ppat.1004192 (PMC4055766; doi:10.1371/journal.ppat.1004192)
Supplement: Table S1 — Clinical scoring used in this study. Animals were monitored during the study to be clinically scored. (DOCX) [file ppat.1004192.s007.docx]

**Table S1. Clinical scoring used in this study**

| **Parameter** | **Degree of parameter** | **Possible score** |
| --- | --- | --- |
| Fever | Normal (< 39 ^o^C) | 0 |
|  | Elevated temperature (39-40 ^o^C) | 3 |
|  | High temperature (> 40 ^o^C) | 5 |
| Posture | Piloerection of body hair | 1 |
|  | Decreased activity, decreasing normal behavior/Occasionally lying down, huddled, active when people in room | 2 |
|  | Huddled on camera, active when people in room/Lying down, getting up when approached, using cage for support | 3 |
|  | Huddled when people in room, shaking, toes and hands clenched/Lying down, not getting up when approached or prompted | 5 |
| Respiration | Increased or decreased; mild cough and clear nasal discharge | 3 |
|  | Labored breathing through mouth; severe cough and severe nasal discharge | 5 |
| Appetite | Slightly decreased | 1 |
|  | Decreased | 2 |
|  | Severely decreased | 5 |
| Skin | Flushed appearance | 2 |
|  | Visible rash | 2 |
|  | Bleeding | 5 |

Animals were monitored every day during the study to be clinically scored. Animals would be euthanized if their clinical scores reached 15 (a humane endpoint).
